# Gravitational Constraints on a Lightlike Boundary

**DOI:** 10.1007/s00023-021-01038-z

**Published:** 2021-03-17

**Authors:** G. Canepa, A. S. Cattaneo, M. Tecchiolli

**Affiliations:** Institut für Mathematik, Universität Zürich, Winterthurerstrasse 190, 8057 Zürich, Switzerland

## Abstract

We analyse the boundary structure of general relativity in the coframe formalism in the case of a lightlike boundary, i.e. when the restriction of the induced Lorentzian metric to the boundary is degenerate. We describe the associated reduced phase space in terms of constraints on the symplectic space of boundary fields. We explicitly compute the Poisson brackets of the constraints and identify the first- and second-class ones. In particular, in the 3+1-dimensional case, we show that the reduced phase space has two local degrees of freedom, instead of the usual four in the non-degenerate case.

## Introduction

The field-theoretical formulation of general relativity (GR) is the assignment to a manifold M of an action functional depending on a Lorentzian metric, whose Euler–Lagrange equations are Einstein’s equations. If we now consider a manifold *M* (of dimension *N*) with boundary $\partial M = \Sigma$, a natural question that can be raised is the structure of the induced data of field equations on the boundary $\Sigma$. This structure can be described through the *reduced phase space* of the theory which encodes the data of the space of boundary fields and of the constraints of the theory.

In this paper, we study the reduced phase space of general relativity (GR) in the coframe formulation in the case where the boundary has a lightlike induced metric. The corresponding geometric structures for the spacelike and timelike cases have already been studied by two of the authors in [5], based on the results outlined in [13]. The differences between the cases are given by the signature of the restriction of the metric to the boundary. Indeed, it turns out that there are major differences between the cases when the metric is *spacelike* or *timelike*—respectively, with signature as a symmetric bilinear form $(N-1,0,0)$ or $(N-2,1,0)$ where the first index denotes positive eigenvalues, the second negative ones and the third zero ones—and when the metric is *lightlike*—with signature $(N-2,0,1)$ where the last entry refers to the transversal direction. Note that, since the metric in the bulk is Lorentzian, the metric on the boundary can only be non-degenerate or have a unique direction along which it is degenerate.

In this paper, following the same scheme of [5, 13], the boundary structure is recovered through a method that was firstly described by Kijowski and Tulczijew (KT) in [20] opposed to the one proposed by Dirac [16]. This latter approach to the problem at hand has been developed in [1]. This article stems from the observations in [5, 13] and describes the geometric structure of the boundary fields by adapting the result to the case of a degenerate boundary metric. In $3+1$ dimensions, this results in a reduced phase space with two local degrees of freedom (in good agreement with the literature [1]) instead of four in the non-degenerate case.^1^

The advantages of the KT alternative, in which the reduced phase space is described as a reduction (i.e. as a quotient space) of the space of free boundary fields, reside principally in the simplification of the procedure that leads to the definition of the constraints starting from the restriction of the Euler–Lagrange equations in the bulk. Furthermore, this construction avoids the introduction of the artificial classifications of the constraints as primary, secondary, etc. Another important virtue of this approach is its compatibility with the BV-BFV construction ([8]), whose quantization procedure ([9]) can then be more easily applied to the theory. The BV-BFV formalism provides a procedure to construct the reduced phase space too; however, it is not applicable in this case for $N\ge 4$ ([13]) since some of the regularity assumptions fail to be satisfied. It is worth noting that the present paper treats only this case, since the case $N=3$ has already been successfully analysed in [6] and does not display the issues of the higher dimensional case.

As mentioned above, in this paper we consider the coframe formulation. More precisely, we use the Palatini–Cartan (PC) formalism (from [7, 21]) since its formulation through differential forms and connection is very convenient for the boundary (and corner [4]) analysis. The choice of the formalism is not immaterial due to the fact that classically equivalent theories on the bulk can behave differently in the presence of a boundary [13, Section 4.3]. This is the case of gravity, where the space of solutions of the Euler–Lagrange equations (modulo symmetries) of the PC and the Einstein–Hilbert formulations are isomorphic, but their Hamiltonian formulations present striking, although classically irrelevant, differences, in particular in the structure of their BV-BFV formalism ( [10, 12]). The Ashtekar formalism provides yet another alternative way through which this problem has been studied in the literature [2, 14]; however, we will not explore this direction. Furthermore, the same problem can be analysed in greater generality such as, for example, the one proposed in [18] (where no compatibility with either the coframe or the internal metric is required) and the parent formulation proposed in [3], but we postpone the comparison with them to future works.

One of the greatest challenges of the constraint analysis of the PC theory comes from the structure of the symplectic form of the *true* space of boundary fields. It is a quotient space of the restriction of the bulk fields to the boundary under an equivalence relation depending on the coframe. Since the use of equivalence classes is usually quite annoying to handle, it is useful to fix a representative and describe the reduced phase space with it. This has been done for a spacelike or timelike boundaries in [5] through the introduction of a suitable *structural constraint*. However, such constraint has to be adapted in the lightlike case, since it fixes the representative only provided that the induced metric on the boundary is degenerate. In this paper, we extend the solution proposed for the space- and timelike cases to a lightlike boundary by considering a suitable adaptation. In particular, the key point is to modify the *structural constraint*. The solution that we find is slightly more involved and gives rise to second class constraints, as opposed to the non-degenerate case where all constraints are first class. The analysis is carried out in full generality for every dimension $N\ge 4$.

Furthermore, we propose a linearized version of the theory, in “Appendix B”, where we work around a reference solution of the Euler–Lagrange equation. In this case, it can be shown that there is a natural isomorphism between the quotient space of the space of fields and another space where no equivalence classes are taken into account. This leads to a large simplification of the computations still retaining some of the key features of the real boundary theory, thus being also a nice toy model for the general case. In order to keep the results as simple and clean as possible, this part has only been developed for $N=4$, but it can be extended without problems to higher dimensions.

The importance of this problem is witnessed by the number of previous works considering the structure of GR on null foliations, the first of which date back to Penrose and Sachs [22, 25]. In particular, the description of the Hamiltonian formulation of GR in the case of a null hypersurface has been studied, for example, in [15, 29] and in [23, 24] in the Einstein–Hilbert formalism . This formulation would allow the construction of exact (but not unique) solutions starting from initial data on null hypersurfaces such as, for example, null horizons of black holes. Furthermore, a Hamiltonian formulation of the theory is widely considered to be one of the best starting points for the quantization of the theory.

### Structure of the Paper

The last sections of this Introduction are devoted to recollecting the background material and reviewing the results of the paper.

In Sect. 2, we state most of the technical results needed throughout the paper. The proofs are collected in “Appendix C” for completeness, but can be skipped by the hasty reader.

The past results and the formal introduction to the problem motivating this work are collected in Sect. 3. In particular, we recall the main results of the non-degenerate case as stated in [5].

Finally, in Sect. 4 we consider the general case and illustrate in full detail the boundary structure of the degenerate case. The main results are collected in Theorem 29.

In “Appendix B”, we develop the corresponding linearized theory which is a simpler toy model of the general case. The structure of the *linearized constraints* is in Theorem 44.

### Palatini–Cartan Formalism

In this section, we present the Palatini–Cartan formalism (see, for example, [27, 28] and references therein for a review of the classical structure) and state the relevant (for our construction) results of [13]. For a more detailed description, we refer to [5, Section 2].

We consider an *N*-dimensional oriented smooth manifold *M* together with a Lorentzian structure so that we can reduce the frame bundle to an $SO(N-1,1)$-principal bundle $P \rightarrow M$. We denote by ${\mathcal {V}}$ the associated vector bundle by the standard representation. Each fibre of ${\mathcal {V}}$ is isomorphic to an *N*-dimensional vector space *V* with a Lorentzian inner product $\eta$ on it. The inner product allows the identification $\mathfrak {so}(N-1,1) \cong \bigwedge ^2 {V}$. Furthermore, we use the shortened notation

1 $$\begin{aligned} \Omega ^{i,j}:= \Omega ^i\left( M, \textstyle {\bigwedge ^j} {\mathcal {V}}\right) \end{aligned}$$

to indicate the spaces of *i*-forms on *M* with values in the *j*th wedge product of ${\mathcal {V}}$.^2^ Moreover, we define the wedge product on these spaces as a map

$$\begin{aligned} \wedge : \Omega ^{i,j} \times&\Omega ^{k,l} \rightarrow \Omega ^{i+k,j+l}&\text {for} \; i+k \le N, \; j+l \le N \\ (\alpha ,&\beta ) \mapsto \alpha \wedge \beta&\end{aligned}$$

by taking the wedge product on both the external ($T^*M$) and internal (${\mathcal {V}}$) parts.^3^ When no confusion can arise, we will omit the wedge symbol and consider it as understood (i.e. any expression of the form $\alpha \beta$ should be interpreted as $\alpha \wedge \beta$).

The dynamical fields of the theory are a *P*-connection $\omega$ and a coframe *e* (a.k.a *N*-bein), i.e. an orientation preserving bundle isomorphism covering the identity

$$\begin{aligned} e :TM {\mathop {\longrightarrow }\limits ^{\sim }}{\mathcal {V}} . \end{aligned}$$

From the coframe, it is possible to recover a metric as

2 $$\begin{aligned} g_{\mu \nu }=\eta (e_\mu ,e_\nu ). \end{aligned}$$

The space of the *P*-connections, denoted with ${\mathcal {A}}(M)$, can be identified, via choosing a reference connection $\omega _0$, to $\Omega ^{1,2}$ thanks to $\mathfrak {so}(N-1,1) \cong \bigwedge ^2 V$. We denote by $d_\omega$ and by $F_\omega \in \Omega ^{2,2}$, respectively, the covariant derivative $\Omega ^{\bullet ,\bullet }\rightarrow \Omega ^{\bullet +1,\bullet }$ associated with a connection $\omega$ and its curvature.

The action functional of the theory is^4^

3 $$\begin{aligned} S = \int _M\left[ \frac{1}{(N-2)!}e^{N-2}F_\omega - \frac{1}{N!}\Lambda e^N\right] \end{aligned}$$

where the notation $e^{k}$ denotes the *k*th wedge power of *e* and $\Lambda$ is a constant (the cosmological constant). From the action, we can deduce the Euler–Lagrange (EL) equations of the theory by taking its variations. The EL equation corresponding to the variation of $\omega$ is $d_\omega (e^{N-2})=0$, and using the Leibniz rule, this equation can be rewritten as $e^{N-3}d_\omega e= 0$, which in turn, as we will see with Lemma 3, is equivalent to

4 $$\begin{aligned} d_\omega e = 0. \end{aligned}$$

The Euler–Lagrange equation corresponding to the variation of *e* is

5 $$\begin{aligned} \frac{1}{(N-3)!}e^{N-3}F_\omega -\frac{1}{(N-1)!}\Lambda e^{N-1}=0. \end{aligned}$$

Equation (4) is the torsion-free condition and identifies the connection $\omega$ with the Levi-Civita connection of the metric (2). With this substitution, (5) corresponds then to the Einstein equations.

### Overview

We present here the problem and the results of the paper at a qualitative level (and for $N=4$) and refer to the subsequent sections for a more precise treatment.

The main contribution of this article, as mentioned in Introduction, is the description of the reduced phase space of general relativity in the PC formalism on lightlike boundaries as the critical locus of functions (or constraints) defined on a symplectic space of boundary fields induced from the bulk structure.

The starting point of this description is the boundary symplectic structure induced by the bulk action following the construction described by [20]. This construction starts from the variation of the classical action and extracts a one-form on the space of the restrictions^5^ of the fields to the boundary. Subsequently, it is possible to get a closed two-form by taking the de Rham differential (on the space of fields) of the original one-form. If this two-form is degenerate, it is then possible to construct a symplectic form^6^ by taking a quotient (under the assumption that the quotient space is smooth). The upshot of the construction in the Palatini–Cartan case, described first in [13] and recalled in detail at the beginning of Sect. 3, is that the symplectic space of the boundary theory is a quotient space $F_{PC}^{\partial }= {\widetilde{F}}_{PC}/_\sim$ where the elements of ${\widetilde{F}}_{PC}$ are the restrictions of the coframe *e* and the connection $\omega$ to the boundary^7^ and the equivalence relation is given by $\omega \sim \omega + v$, with *v* satisfying $e \wedge v=0$. The resulting symplectic form is

$$\begin{aligned} {\varpi _{PC}^{\partial } }= \int _{\Sigma } e \delta e \delta [\omega ]. \end{aligned}$$

Now, in order to pass from the symplectic space of boundary fields, or *geometric phase space*, to the *reduced phase space*, we must identify the correct constraints of the theory. The natural candidates for the constraints on the boundary are the restrictions of the Euler–Lagrange equations that contain no derivatives transversal to the boundary

$$\begin{aligned} d_\omega e = 0 \quad \text {and} \quad e F_\omega -\frac{1}{6}\Lambda e^{3}=0. \end{aligned}$$

However, these functions are not invariant under the change of representative in the aforementioned quotient space. Indeed, let us consider the first equation and consider two different $\omega \sim \omega '$, i.e. $\omega = \omega '+v$ with $ev=0$. The equation $d_\omega e = 0$ does not necessarily imply $d_{\omega '} e = 0$ since we get an additional term: $d_\omega e = d_{\omega '} e + [v,e]$ and in general $[v,e]\ne 0$ for $v \in \Omega _{\partial }^{1,2}$ such that $ev=0$.

In [5], a convenient solution was found in the case of non-degenerate boundary metric, whereas in [13] a general solution is outlined. The object of this paper is to find an analogous solution in the degenerate case and therefore to generalize the result of [5, 13] to all possible boundary metrics.

The construction of the non-degenerate case is described in detail in Sect. 3 and consists on imposing an equation fixing a convenient representative of the equivalence class $[\omega ]$:

6 $$\begin{aligned} e_n d_{\omega } e \in {{\,\mathrm{Im}\,}}( e \wedge \cdot ). \end{aligned}$$

Here, $e_n \in \Omega _{\partial }^{0,1}$ is a field linearly independent from the tangent components of *e* restricted to the boundary.^8^ The rationale behind this condition is to partially reobtain a condition on bulk fields that is not transferred to the boundary fields. Indeed, one of the EL equations ($ed_{\omega }e=0$, in the bulk equivalent to $d_{\omega }e=0$) can be written in a neighbourhood of the boundary as an evolution equation: $e_n d_{\omega }e + e \partial _n e + e[\omega _n,e]+ e d_{\omega }e_n=0$ where the index *n* denotes a component transversal to the boundary. It is then easy to see that since the last terms are all in the image of $e \wedge \cdot$, also the first term must be in this space. We can then use this condition on the boundary to fix the representative of the class $[\omega ]$ (see Sect. 2 for the notation and Theorem 15 for the details). We call this condition the *structural constraint*.^9^

Using the representative fixed by (6), it is then possible to write a set of constraints generating the same critical locus of the original ones and which are invariant as follows:

$$\begin{aligned} L_c&= \int _{\Sigma } c e d_{\omega } e, \\ P_{\xi }&= \int _{\Sigma } \iota _{\xi } e e F_{\omega } + \iota _{\xi } (\omega -\omega _0) e d_{\omega } e, \\ H_{\lambda }&= \int _{\Sigma } \lambda e_n \left( e F_{\omega } +\frac{1}{3!}\Lambda e^{3} \right) \end{aligned}$$

where *c*, $\xi$ and $\lambda$ are suitable Lagrange multipliers. A very important bit of information is given by the structure of their Poisson brackets which is collected in Theorem 18 and shows that these constraints are first-class.

This solution, and in particular the choice of the structural constraint, requires that the induced metric $g^\partial = e^{*}\eta$ be non-degenerate and does not work in the degenerate case. The adaptation of such approach to the degenerate case is the object of this paper, and in the following paragraphs, we will give an overview on how to overcome the differences of this case.

#### Remark 1

In this paper, we address the problem assuming that in the boundary manifold there exists a lightlike subset and we assume to be working only in an open subset of the lightlike one. The general case of a boundary with points of different types (lightlike, spacelike and timelike) can be recovered as explained in Remark 2.

The main difference in the degenerate case is the impossibility of finding a representative of the equivalence class $[\omega ]$ satisfying the structural constraint. The idea is to modify this equation by subtracting the problematic part and impose a weakened structural constraint as follows:

7 $$\begin{aligned} e_n d_{\omega } e - e_n p_{{\mathcal {T}}}(d_{\omega } e) \in {{\,\mathrm{Im}\,}}( e \wedge \cdot ) \end{aligned}$$

where $p_{{\mathcal {T}}}$ is the projection to an appropriately defined subspace (see (9); see also Sect. 2 for the notation and Theorem 19 for more details). This weakened structural constraint no longer fixes the representative in the equivalence class uniquely, and hence, it has to be supplemented with another set of equations, though of little importance for the construction. Furthermore, this weakened constraint does not guarantee the equivalence between the constraint $L_c$ and $d_{\omega }e=0$. Indeed, an important feature that was a key point in the non-degenerate case was the fact that the equation $ed_{\omega }e=0$, after imposing the structural constraint $e_n d_{\omega }e= \in {{\,\mathrm{Im}\,}}( e \wedge \cdot )$, defines the same zero locus as $d_{\omega } e=0$. As a consequence, in order to get the correct reduced phase space, in the degenerate case one has to add an additional constraint accounting for the missing part in the weakened structural constraint: namely,

$$\begin{aligned} R_{\tau } = \int _{\Sigma } \tau d_{\omega } e \end{aligned}$$

with $\tau$ belonging to an appropriate space ${\mathcal {S}}$(see (9c) for the definition). We will call this constraint the *degeneracy constraint*.^10^ This construction is made precise in the first part of Sect. 4 where we also analyse the structure of this new set of constraints (Theorem 29 and Corollary 33).

By computing the Poisson brackets of the constraints, we show that all the constraints are first class except the degeneracy constraint $R_{\tau }$ which is second class. Finally, we also compute the number of local physical degrees of freedom of the theory. In dimension 3+1, we obtain that the reduced phase space has two local degrees of freedom.

#### Remark 2

This construction can be extended to the general case of a boundary only part of which is allowed to be lightlike. In this case, the field $\tau \in {\mathcal {S}}$ defining the degeneracy constraint has support in the closure of the lightlike points. Furthermore, since the equations defining $\tau \in {\mathcal {S}}$ are algebraic, by continuity we also have that $\tau$ vanishes on the boundary (if present) of the closed lightlike subset.

The linearized theory follows a similar pattern. It retains the most important properties of the general theory (e.g. the number of physical local degrees of freedom) and can be therefore thought of as an interesting toy model of the latter. The complete analysis of this case has been detailed in “Appendix B”. Furthermore, the linearized case is treated in the physical case $N=4$ only, hence providing a simple reference for the formulas and results in this case.

**Table 1:**
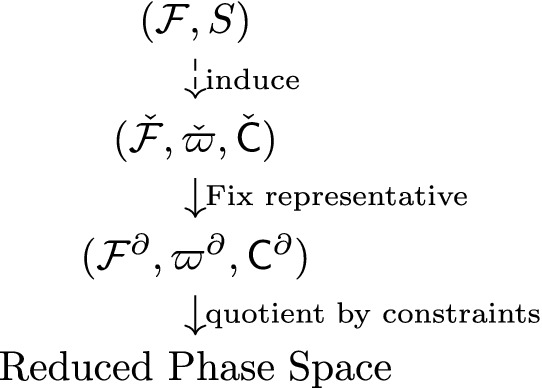
Step by step construction of the reduced phase space

We can recollect the steps in Table 1. The starting point is the bulk structure, given by the space of fields ${\mathcal {F}}$ and the action *S*. Then, we induce a preboundary structure $(\check{{\mathcal {F}}}, {\check{\varpi }}, \check{{\mathsf {C}}})$ where $\check{\mathsf {C}}$ represents the restriction of the EL equations to the boundary. Subsequently, we fix a representative in the equivalence class of $[\omega ]$ and obtain the geometric phase space $({\mathcal {F}}^{\partial }, {\varpi }^{\partial })$ where the constraints ${\mathsf {C}}^{\partial }$ are well defined. Finally, the reduced phase space is obtained as the quotient of the geometric phase space by the constraints.

**Table 2:** Differences between the non-degenerate case and the lightlike one

	Non-degenerate case	Lightlike case
Geometric phase space	$({\mathcal {F}}^{\partial }, \varpi ^{\partial })$	$({\mathcal {F}}^{\partial }, \varpi ^{\partial })$
Structural constraint	(6)	(7)
Constraints	$L_c, P_{\xi }, H_{\lambda }$	$L_c, P_{\xi }, H_{\lambda }, R_{\tau }$

We conclude the overview with Table 2 showing the differences between the non-degenerate case and the lightlike one.

## Technical Results

In this section, we define the relevant quantities and maps, establish the conventions and summarize the technical results needed in the paper. One of the goal of this section is to prove some mathematical results in order to make the subsequent construction more fluid and easy to read. Full proofs and detailed computations will be postponed to “Appendix C”.

We first recall and introduce some useful shorthand notation. We will denote by $\Sigma = \partial M$ the $(N-1)$-dimensional boundary of the manifold *M* of dimension *N*. Furthermore, we will use the notation ${\mathcal {V}}_{\Sigma }$ for the restriction of ${\mathcal {V}}$ to $\Sigma$. Extending the notation introduced in (1), using the same conventions, we also define

$$\begin{aligned} \Omega ^{i,j}:= \Omega ^i\left( M, \textstyle {\bigwedge ^j} {\mathcal {V}}\right) \qquad \Omega _{\partial }^{i,j}:= \Omega ^i\left( \Sigma , \textstyle {\bigwedge ^j} {\mathcal {V}}_{\Sigma }\right) . \end{aligned}$$

We define the number of degrees of freedom of the space $\Omega ^{i,j}$ (and $\Omega _{\partial }^{i,j}$) as its dimension as a $C^\infty$-module. We will sometimes simply denote this by *dimension*.

The coframe *e* viewed as an isomorphism $e:TM \rightarrow {\mathcal {V}}$ defines, given a set of coordinates on *M*, a preferred basis on ${\mathcal {V}}$. If we denote by $\partial _i$ the vector field in *TM* corresponding to the coordinate $x_i$, we get a basis on ${\mathcal {V}}$ by $e_i:= e (\partial _i)$. On the boundary, since $T\Sigma$ has one dimension less than ${\mathcal {V}}_{\Sigma }$, we can complement the linear independent set $e_i$ with another independent vector that we will call $e_n$. We call this basis the *standard basis* (this basis depends on a given coordinate system on *M* (or $\Sigma$)) and, unless otherwise stated, the components of the fields will always be taken with respect to this basis.

On $\Omega ^{i,j}$ and $\Omega _{\partial }^{i,j}$, we define the following maps:

$$\begin{aligned} W_{k}^{ (i,j)}: \Omega ^{i,j}&\longrightarrow \Omega ^{i+k,j+k} \\ X&\longmapsto X \wedge \underbrace{e \wedge \dots \wedge e}_{k-times}, \\ W_{k}^{ \partial , (i,j)}: \Omega _{\partial }^{i,j}&\longrightarrow \Omega _{\partial }^{i+k,j+k}\\ X&\longmapsto X \wedge \underbrace{e \wedge \dots \wedge e}_{k-times}. \end{aligned}$$

Recall that the elements of the Lie algebra $\mathfrak {so}(N-1,1)$ can be identified with the elements of $\Omega ^{(0,2)}$ (or $\Omega _{\partial }^{(0,2)}$, depending on where we consider such elements). Hence, the Lie brackets define a map

$$\begin{aligned}{}[\cdot , \cdot ] : \Omega ^{(0,2)} \times \Omega ^{(0,2)}&\rightarrow \Omega ^{(0,2)} \\ (x,y)&\mapsto [x,y], \end{aligned}$$

and a similar one on $\Omega _{\partial }^{(0,2)}$. Combining this action with the wedge product, we can define the following generalisation, denoted with the same symbol

$$\begin{aligned}{}[\cdot , \cdot ] : \Omega ^{(i,2)} \times \Omega ^{(k,2)}&\rightarrow \Omega ^{(i+k,2)}&\text {for} \; i+k \le N\\ (x,y)&\mapsto [x,y], \end{aligned}$$

which in coordinates reads

$$\begin{aligned}{}[x,y]_{\mu _1 \dots \mu _{i+k}}^{a_1 a_2} = \sum _{\sigma _{i+k}} \text {sign}(\sigma _{i+k}) x_{\mu _{\sigma (1)} \dots \mu _{\sigma (i)}}^{a_1 a_3} y_{\mu _{\sigma (i+1)} \dots \mu _{\sigma (i+k)}}^{a_2 a_4}\eta _{a_3 a_4}. \end{aligned}$$

Furthermore, generalizing the action of the Lie algebra $\mathfrak {so}(N-1,1)$ on ${\mathcal {V}}$ (or ${\mathcal {V}}_{\Sigma }$) we can also introduce the following maps:

8 $$\begin{aligned} \varrho ^{(i,j)} : \Omega _{\partial }^{i,j}&\longrightarrow \Omega _{\partial }^{i+1,j-1} \\ X&\longmapsto [X, e] . \nonumber \end{aligned}$$

In coordinates, they are defined as

$$\begin{aligned} X&\mapsto \sum _{\sigma _{i+1}} \text {sign}(\sigma _{i+1}) X_{\mu _{\sigma (1)}\dots \mu _{\sigma (i)}}^{a_{1} \dots a_{j}} \eta _{a_{j} b} e^{b}_{\mu _{\sigma (i+1)}}. \end{aligned}$$

In the next part of this section, we will state some technical results. We refer to Appendix of [5] for fully exhaustive proofs. As in the aforementioned article, we use by convention the total degree^11^ to fix the commutation relations between quantities in $\Omega ^{i,j}$ and $\Omega _{\partial }^{i,j}$. For example, given two elements^12^$\alpha \in \Omega ^{i,j}$ and $\beta \in \Omega ^{k,l}$ of total degree $i+j$ and $k+l$, respectively, we have the following commutation rule:

$$\begin{aligned} \alpha \beta = (-1)^{(i+j)(k+l)}\beta \alpha . \end{aligned}$$

The properties of the maps $W_{k}^{ (i,j)}$ and $W_{k}^{ \partial , (i,j)}$ do not depend on the degeneracy of $g^{\partial }$. Hence, we have the following results ([5, 13]):

### Lemma 3

Let $$N=\mathrm {dim}(M)\ge 4$$. Then,

1. $$ W_{N-3}^{ (2,1)}$$ is bijective;
2. $$ \mathrm {dim}\mathrm {Ker}W_{N-3}^{(2,2)}\not =0$$.

### Lemma 4

The maps $W_{k}^{ \partial , (i,j)}$ have the following properties for $N \ge 4$:

1. $W_{N-3}^{\partial , (2,1)}$ and $W_{N-3}^{\partial , (1,2)}$ are surjective but not injective;
2. $$W_{N-3}^{\partial , (1,1)}$$ is injective;
3. $$\dim \mathrm {Ker} W_{N-3}^{\partial , (1,2)} = \dim \mathrm {Ker} W_{N-3}^{\partial , (2,1)}$$;
4. $$W_{N-4}^{\partial , (2,1)}$$ is injective. ($$N \ge 5$$)

The following lemma is an extension of the corresponding ones in [5] and in [13]. All the proofs of the following results can be found in “Appendix C”.

### Lemma 5

If $g^\partial$ is degenerate with $\dim \mathrm {Ker}{(g^\partial )}=1$, then $\varrho ^{(1,2)} |_{\mathrm {Ker} W_{N-3}^{\partial , (1,2)}}$ has a kernel of dimension $\frac{N(N-3)}{2}$.

### Remark 6

These three lemmas express in a mathematical way the possibility of inverting the coframe *e* when appearing in a wedge product or in the generalised Lie algebra action $\varrho$ of (8). In particular (exemplifying only in dimension $N=4$), they give the answer to the following question: given an expression of the form $e\wedge X$ or [*e*, *X*] for some *X*, is it possible to invert these expressions and get back *X*? The answer is that it depends on the space where *X* is defined, and in the case of $\varrho$ on the degeneracy of the boundary metric $g^\partial$. For example, if we have $X \in \Omega ^{2,1}$, using Lemma 3, we see that it is possible to define an inverse $``W^{-1}_1"$ such that $X= W^{-1}_1(e \wedge X)$. On the contrary, for $X \in \Omega _{\partial }^{2,1}$ , using Lemma 4, such inversion is no longer possible in a unique way, meaning that $e\wedge X$ does not contain all the information that *X* contained (or, said in another way, not all the components of *X* appear in $e \wedge X$). Note also that these maps do not appear in the three-dimensional case. Hence, their properties give hints on the differences between the topological three-dimensional theory and the physical four-dimensional one.

### Results for the Degeneracy Constraint

In order to define the space to which the Lagrange multiplier of the degeneracy constraint belongs, it is useful to consider the following construction.

If a metric $g^{\partial }$ is degenerate, we can find a vector field *X* on $\Sigma$ such that $\iota _{X}g^{\partial }=0$. Using a reference metric $g_0$, we can complete the vector field $X_0$ (with $\iota _{X_0}g_0^{\partial }=0$) to a basis $X_0, Y_0^i$ of *TM*. If we then choose a coframe *e* *near* the original one, the same $Y^i_0$s would also be a completion of *X* to a basis of *TM*.

Let now $\beta \in \Omega _{\partial }^{1,0}$ a one form such that $\iota _X \beta =1$. We then define ${\widehat{e}}= \beta \iota _X e$ and fix $\beta$ by requiring that ${\widetilde{e}} {:=} e - {\widehat{e}}$ satisfies^13^

$$\begin{aligned} \iota _{Y_0^1} \dots \iota _{Y_0^{N-2}}({\widetilde{e}} \wedge e^{N-4} \wedge v )= 0 \end{aligned}$$

for all $v\in \Omega _{\partial }^{1,2}$ such that $e^{N-3} \wedge v = 0$. Using this notation, we can define another set of maps

$$\begin{aligned} {\widetilde{\varrho }}^{(i,j)} : \Omega _{\partial }^{i,j}&\longrightarrow \Omega _{\partial }^{i+1,j-1} \\ X&\longmapsto [X, {\widetilde{e}}] \end{aligned}$$

which in coordinate reads

$$\begin{aligned} X&\mapsto \sum _{\sigma _{i+1}} \text {sign}(\sigma _{i+1}) X_{\mu _{\sigma (1)}\dots \mu _{\sigma (i)}}^{a_{1} \dots a_{j}} \eta _{a_{j} b} {\widetilde{e}}^{b}_{\mu _{\sigma (i+1)}}. \end{aligned}$$

Let *J* be a complement^14^ of the space ${{\,\mathrm{Im}\,}}\varrho ^{(1,2)} |_{\mathrm {Ker} W_{N-3}^{\partial , (1,2)}}$ in $\Omega _{\partial }^{2,1}$. We now consider the following spaces:

9a $$\begin{aligned} {\mathcal {T}}&= \mathrm {Ker}W_{N-3}^{\partial (2,1)} \cap J \subset \Omega _{\partial }^{2,1}, \end{aligned}$$

9b $$\begin{aligned} {\mathcal {K}}&= \mathrm {Ker}W_{N-3}^{\partial (1,2)} \cap \mathrm {Ker} \varrho ^{(1,2)} \subset \Omega _{\partial }^{1,2}, \end{aligned}$$

9c $$\begin{aligned} {\mathcal {S}}&= \mathrm {Ker}W_{1}^{\partial (N-3,N-1)} \cap \mathrm {Ker} {\widetilde{\varrho }}^{(N-3,N-1)} \subset \Omega ^{N-3,N-1}_{\partial }. \end{aligned}$$

#### Remark 7

Note that all these three spaces are zero in the non-degenerate case. In particular, the fact that ${\mathcal {K}}$ is not zero in the degenerate case accounts for the existence of components of $\omega$ that do not appear either in the expression $ed_{\omega }e$ or $e_n d_{\omega }e$ but do appear in $d_{\omega }e$ (for $N=4$). Hence, ${\mathcal {K}}$ represents the failure of the structural constraint to fix uniquely a representative in the equivalence class $[\omega ]$. The space ${\mathcal {T}}$ is strictly related to ${\mathcal {K}}$ since it contains elements of $\mathrm {Ker}W_{N-3}^{\partial (2,1)}$ that cannot be generated by elements in $\mathrm {Ker}W_{N-3}^{\partial (1,2)}$ through $\varrho ^{(1,2)}$. As a matter of fact, using coordinates, one can see that the components of $\Omega _{\partial }^{2,1}$ corresponding to ${\mathcal {T}}$ in the non-degenerate case are generated through $\varrho ^{(1,2)}$ by the elements corresponding to ${\mathcal {K}}$ in $\Omega _{\partial }^{1,2}$. Finally, ${\mathcal {S}}$ plays the role of the dual of ${\mathcal {T}}$ as specified in Lemma 10.

We also denote by $p_{{\mathcal {T}}}: \Omega _{\partial }^{2,1} \rightarrow {\mathcal {T}}$, by $p_{{\mathcal {K}}}: \Omega _{\partial }^{1,2} \rightarrow {\mathcal {K}}$ and by $p_{{\mathcal {S}}}: \Omega _{\partial }^{N-3,N-1} \rightarrow {\mathcal {S}}$ some corresponding projections to them.^15^ The spaces ${\mathcal {T}}$ and ${\mathcal {K}}$ are not empty because of the results of Lemmas 4.(1) and 5 , while ${\mathcal {S}}$ is characterized by the following proposition in which we also summarize the involved components, since they will be crucial in the computation of the Poisson brackets of the constraints.

#### Proposition 8

The dimension of ${\mathcal {S}}$ is

$$\begin{aligned} \dim {\mathcal {S}} = \frac{N(N-3)}{2}. \end{aligned}$$

Let $p\in \Sigma$ and *U* a neighbourhood of *p* in which normal coordinates centred in *p* are well defined. Then, using such coordinates and the standard basis of ${\mathcal {V}}_{\Sigma }$, the nonzero components of an element $\tau \in {\mathcal {S}}$ are

$$\begin{aligned} Y_{\mu }&:=\tau ^{NN-1 \mu _1 \dots \mu _{N-3}}_{\mu _1 \dots \mu _{N-3}} \text { where } \mu \ne \mu _1 \dots \mu _{N-3}, \\ X_{\mu _1}^{\mu _2}&:= \tau ^{N N-1 \mu _3 \dots \mu _{N-2} \mu _{2}}_{\mu _3 \dots \mu _{N-2} \mu _1}, \end{aligned}$$

satisfying

$$\begin{aligned} \sum _{\mu =1}^{N-2} Y_{\mu } =0 \text { and } X_{\mu _1}^{\mu _2} = f({\widetilde{g}}^{\partial }, X_{\mu _2}^{\mu _1}, Y_{\mu }) \end{aligned}$$

for $\mu _1 < \mu _2$ and some linear function *f* with ${\widetilde{g}}^{\partial } {:=} \eta ({\widetilde{e}},{\widetilde{e}})$.

The proof of this Proposition is postponed to “Appendix C”.

#### Remark 9

In order to compute the structure of the Poisson brackets between the constraints, we will need to know the equations defining ${\mathcal {S}}$ not only point-wise but also in a small neighbourhood, since we will need to take derivatives. Despite being in principle computable for every dimension, we do not need the explicit expression of *f*. It is also worth noting that in the base point *p* of the normal coordinates, the last set of equations reduces to

$$\begin{aligned} X_{\mu _1}^{\mu _2} =- X_{\mu _2}^{\mu _1}. \end{aligned}$$

While the spaces ${\mathcal {K}}$ and ${\mathcal {T}}$ arise naturally while considering the symplectic reduction of the boundary two-form, the importance of the space ${\mathcal {S}}$ resides in the following proposition that shows that ${\mathcal {S}}$ plays the role of a *dual space* of ${\mathcal {T}}$.

#### Lemma 10

Let $$\alpha \in \Omega ^{2,1}_\partial $$. Then,

$$\begin{aligned} \int _{\Sigma } \tau \alpha =0 \,\, \forall \tau \in {\mathcal {S}} \Longrightarrow p_{{\mathcal {T}}}(\alpha )=0. \end{aligned}$$

We conclude this section with a result that will be necessary in the computation of the Hamiltonian vector fields of the constraints and in their Poisson brackets.

#### Lemma 11

$$\begin{aligned} {{\,\mathrm{Im}\,}}\varrho ^{(N-1,N-3)}|_{{\mathcal {S}}} \subset {{\,\mathrm{Im}\,}}W_{N-3}^{\partial , (1,1)} . \end{aligned}$$

#### Corollary 12

The free components of $W_{N-3}^{-1}([\tau ,e])$ are

$$\begin{aligned}{}[W_{N-3}^{-1}([\tau ,e])]_{\mu _1}^{\mu _2}&\propto X_{\mu _1}^{\mu _2} \\ [W_{N-3}^{-1}([\tau ,e])]_{\mu }^{\mu }&\propto Y_{\mu } \end{aligned}$$

such that $\sum _{\mu =1}^{N-2} [W_{N-3}^{-1}([\tau ,e])]_{\mu }^{\mu }=0$ and $[W_{N-3}^{-1}([\tau ,e])]_{\mu _1}^{\mu _2}=-[W_{N-3}^{-1}([\tau ,e])]_{\mu _2}^{\mu _1}$.

The proofs of these lemmas and of the corollary are collected in “Appendix C”.

## Boundary Structure and Known Results

In this section, we give an overview about the symplectic boundary structure of Palatini–Cartan gravity induced from the bulk using the construction introduced by Kijowski and Tulczijew [20]. In other words, we give a description of the *geometric phase space*, i.e. the natural space of fields associated with the boundary before imposing the constraints, and describe the symplectic reduction that produces the reduced phase space. Referring to Table 1 in the overview, we give information about the first step $({\mathcal {F}},S) \rightarrow (\check{{\mathcal {F}},{\check{\varpi }}},\check{{\mathsf {C}}})$ and about the geometric phase space. This part is common to both the non-degenerate (spacelike or timelike) case and degenerate cases (lightlike).

We dedicate this section to the common framework of the two cases and to the non-degenerate one by recalling the most important steps and results. This will be particularly useful, since the analysis of the degenerate case will start from these results trying to solve the various issues arising from the different structural constraints that we will choose. In particular, the crucial difference will come from the different outcome of Lemma 5 in the degenerate and non-degenerate cases.

The investigation of the Hamiltonian formulation follows, as explained before, the construction introduced by Kijowski and Tulczijew [20]. The starting point is the description of what we call *geometric phase space* ${F}^{\partial }_{PC}$. This step is fully detailed in [13]. We consider the restriction of the fields *e* and $\omega$ to the boundary $\Sigma$ and reinterpret them, respectively, as an injective bundle map $T\Sigma \rightarrow {\mathcal {V}}_{\Sigma }$ (that we will call *boundary coframe*) and an orthogonal connection associated with ${\mathcal {V}}_{\Sigma }$. We call ${\widetilde{F}}_{PC}$ the space of these fields, i.e. the space of the restriction of the bulk fields to the boundary. The key point of the construction is to define a one-form on the space ${\widetilde{F}}_{PC}$ as the boundary term arising from the variation of the action through the formula

$$\begin{aligned} \delta S = \mathcal {EL} + \pi ^* {\check{\alpha }} \end{aligned}$$

where $\mathcal {EL}$ are the parts defining the Euler–Lagrange equation and $\pi$ is the restriction to the boundary.

In our case, we get

$$\begin{aligned} {\check{\alpha }} = { \frac{1}{(N-2)!}}\int _{\Sigma } e^{N-2}\delta \omega . \end{aligned}$$

From this one-form it is possible to construct a closed two-form by applying the de Rham differential $\delta$ (of the space of fields):

$$\begin{aligned} {\check{\varpi }}= \delta {\check{\alpha }} = { \frac{1}{(N-3)!}}\int _{\Sigma } e^{N-3}\delta e\delta \omega . \end{aligned}$$

This two-form is a candidate to be a symplectic form on the space of boundary fields; however, it is degenerate, since the function $W_{N-3}^{\partial , (1,1)}$ has a nonzero kernel (Lemma 4): the kernel is parametrized by the vector fields $X= v \frac{\delta }{\delta \omega } \in {\mathfrak {X}}({\widetilde{F}}_{PC})$ with *v* such that

10 $$\begin{aligned} e^{N-3} v=0. \end{aligned}$$

In order to get a symplectic form, we can perform a symplectic reduction by quotienting along the kernel. The *geometric phase space* of boundary fields, determined by the reduction

11 $$\begin{aligned} \pi _{PC}:{\widetilde{F}}_{PC} \longrightarrow {F}^{\partial }_{PC}, \end{aligned}$$

is then parametrized by the field *e* and by the equivalence classes of $\omega$ under the relation $\omega \sim \omega + v$ with *v* satisfying (10). We denote by ${\mathcal {A}}^{red}(\Sigma )$ the space of such equivalence classes. Then, the symplectic form on ${F}^{\partial }_{PC}$ is given by

12 $$\begin{aligned} \varpi ^{\partial }_{PC} = \int _{\Sigma } e^{N-3} \delta e \delta [\omega ] \end{aligned}$$

where we dropped the unimportant prefactor $\frac{1}{(N-3)!}$.

The symplectic space $({F}^{\partial }_{PC},\varpi ^{\partial }_{PC})$ is the space on which we can define the constraints and subsequently perform a reduction over them to get the reduced phase space. The constraints are now to be recovered from the restriction of the Euler–Lagrange equation on the bulk to the boundary. In particular, we have to consider those equations not containing derivatives in the transversal direction, i.e. the evolution equations.

However, some obstruction might occur. We performed a reduction to get the symplectic form (12), yet the restriction of the functions whose zero-locus defines the Euler–Lagrange equations might not be basic with respect to it, i.e. it might not be possible to write such restrictions in terms of the variables of the reduced symplectic space ${F}^{\partial }_{PC}$. This is exactly what happens in our case: a simple check shows that the candidates to be the constraints coming from (5) are not invariant under the transformation $\omega \mapsto \omega +v$. The way out proposed in [5] for the non-degenerate case is to fix a convenient representative of the equivalence class $[\omega ]$ and work out the details with it. In the next section, we will recap the strategy and present the most important steps. This will turn to be useful also in the degenerate case.

### Non-degenerate Boundary Metric

We recall here the steps to get the reduced phase space in the non-degenerate case as developed in [5]. We refer to this work for the proofs and details that are omitted here.

As already mentioned, we define $e_n$ as a section of ${\mathcal {V}}_{\Sigma }$ that is a completion of the basis $e_1,e_2,\dots , e_{N-1}$. Then, we have the following two results:

#### Lemma 13

Let now $g^{\partial }$ be non-degenerate and let $\alpha \in \Omega ^{2,1}_\partial$. Then, $\alpha =0$ if and only if

13 $$\begin{aligned} {\left\{ \begin{array}{ll} e^{N-3}\alpha =0 \\ e_n e^{N-4}\alpha \in {{\,\mathrm{Im}\,}}W_{N-3}^{\partial , (1,1)} \end{array}\right. }. \end{aligned}$$

#### Lemma 14

Let $\beta \in \Omega ^{N-2,N-2}_\partial$. If $g^\partial$ is non-degenerate, there exists a unique $v \in \mathrm {Ker} W_{N-3}^{\partial , (1,2)}$ and a unique $\gamma \in \Omega _{\partial }^{1,1}$ such that

$$\begin{aligned} \beta = e^{N-3} \gamma + e_n e^{N-4} [v, e]. \end{aligned}$$

The key idea is to use these results to fix a representative for the equivalence class $[\omega ] \in {\mathcal {A}}^{red}(\Sigma )$ appearing in the symplectic form (12). Applying Lemma 13 to $\alpha = d_\omega e$, we get that the constraint (coming from the bulk) $d_\omega e = 0$ can be divided into the *invariant constraint* $e^{N-3} d_\omega e = 0$ and the constraint

14 $$\begin{aligned} e_n e^{N-4} d_{\omega } e \in {{\,\mathrm{Im}\,}}W_{N-3}^{\partial ,(1,1)}, \end{aligned}$$

called *structural constraint*. Then, the following results prove that (14) exactly fixes a representative of the aforementioned equivalence class without imposing further constraints.

#### Theorem 15

([5]). Suppose that $g^{\partial }$, the metric induced on the boundary, is non-degenerate. Given any ${\widetilde{\omega }} \in \Omega _{\partial }^{1,2}$, there is a unique decomposition

15 $$\begin{aligned} {\widetilde{\omega }}= \omega +v \end{aligned}$$

with $\omega$ and *v* satisfying

16 $$\begin{aligned} e^{N-3}v=0 \quad \text { and } \quad e_n e^{N-4} d_{\omega } e \in {{\,\mathrm{Im}\,}}W_{N-3}^{\partial ,(1,1)}. \end{aligned}$$

#### Corollary 16

The field $\omega$ in the decomposition (15) depends only on the equivalence class $[\omega ] \in {\mathcal {A}}^{red}(\Sigma )$.

Having fixed the representative of the equivalence class of the connection, one considers the restriction of the Euler–Lagrange equations to the boundary to get the corresponding constraints. The wise choice of the structural constraint (14) allows to construct the set of constraints on the boundary. Defining $c \in \Omega ^{0,2}_\partial [1]$, $\xi \in {\mathfrak {X}}[1](\Sigma )$ and $\lambda \in \Omega ^{0,0}_\partial [1]$ as (odd)^16^ Lagrange multipliers, they read

17a $$\begin{aligned}&L_c = \int _{\Sigma } c e^{N-3} d_{\omega } e , \end{aligned}$$

17b $$\begin{aligned}&P_{\xi }= \int _{\Sigma } \iota _{\xi } e e^{N-3} F_{\omega } + \iota _{\xi } (\omega -\omega _0) e^{N-3} d_{\omega } e, \end{aligned}$$

17c $$\begin{aligned}&H_{\lambda } = \int _{\Sigma } \lambda e_n \left( e^{N-3} F_{\omega } +\frac{1}{(N-1)!}\Lambda e^{N-1} \right) , \end{aligned}$$

where $\omega _0$ is a reference connection.^17^

#### Remark 17

We use here odd Lagrange multipliers *c*, $\xi$ and $\lambda$, following [5]. The notation [1] next to the symbol of the space to which these quantities belong denotes the shift to odd quantities. This convention does not modify the structure of the constraints and simplifies the computations and the notation. The version with even Lagrange multipliers can be easily derived from the present one. For example, let us consider $\{L_c,L_c\}$. This bracket denotes an antisymmetric quantity in which the odd variables are space holders. This means that going back to unshifted (i.e. even) variables, say, $\alpha ,\beta$, a formula like

$$\begin{aligned} \{L_c,L_c\}=-\frac{1}{2} L_{[c,c]} \end{aligned}$$

simply means

$$\begin{aligned} \{L_\alpha ,L_\beta \} =- L_{[\alpha ,\beta ]}. \end{aligned}$$

The following theorem describes the structure of the constraints:

#### Theorem 18

([5]). Let $g^\partial$ be non-degenerate on $\Sigma$. Then, the functions $L_c$, $P_{\xi }$, $H_{\lambda }$ are well defined on ${F}^{\partial }_{PC}$ and define a coisotropic submanifold with respect to the symplectic structure $\varpi ^{\partial }_{PC}$. In particular, they satisfy the following relations

18a $$\begin{aligned} \{L_c, L_c\} = - \frac{1}{2} L_{[c,c]}&\quad \{P_{\xi }, P_{\xi }\} = \frac{1}{2}P_{[\xi , \xi ]}- \frac{1}{2}L_{\iota _{\xi }\iota _{\xi }F_{\omega _0}} \end{aligned}$$

18b $$\begin{aligned} \{L_c, P_{\xi }\} = L_{{\mathcal {L}}_{\xi }^{\omega _0}c}&\quad \{L_c, H_{\lambda }\} = - P_{X^{(a)}} + L_{X^{(a)}(\omega - \omega _0)_a} - H_{X^{(n)}} \end{aligned}$$

18c $$\begin{aligned} \{H_{\lambda },H_{\lambda }\} =0&\quad \{P_{\xi },H_{\lambda }\} = P_{Y^{(a)}} -L_{ Y^{(a)} (\omega - \omega _0)_a} +H_{ Y^{(n)}} \end{aligned}$$

where

$$\begin{aligned} {\mathcal {L}}_{\xi }^{\omega } A = \iota _{\xi } d_{\omega } A - d_{\omega } \iota _{\xi } A \qquad A \in \Omega ^{i,j}_{\partial } \end{aligned}$$

and $X= [c, \lambda e_n ]$, $Y = {\mathcal {L}}_{\xi }^{\omega _0} (\lambda e_n)$ and $Z^{(a)}$, $Z^{(n)}$ are the components of $Z\in \{X,Y\}$ with respect to the frame $(e_a, e_n)$.^18^

## Degenerate Boundary Structure

In Sect. 3, we presented the construction of the boundary structure in the non-degenerate case. Let now $g^{\partial }$ be degenerate, i.e. admitting a vector field *X* such that $\iota _X g^{\partial }=0$.

### Fixing a Representative

In this section, we describe a possible way for fixing the freedom of the choice of the connection $\omega \in [\omega ]$ , adapting the non-degenerate case presented in [5] and summarized in Sect. 3.1. The main difference is that in the degenerate case, because of the different outcome of Lemma 5, it is no longer possible to find an $\omega \in [\omega ]$ such that $e_n e^{N-4}d_{\omega } e \in {{\,\mathrm{Im}\,}}W_{N-3}^{\partial ,(1,1)}$. Indeed, in contrast to the non-degenerate case, the map

$$\begin{aligned} v \in \mathrm {Ker}{(W_{N-3}^{\partial ,(1,2)} )}\mapsto e_n e^{N-4} [v,e] \in \Omega _{\partial }^{N-2,N-2} \end{aligned}$$

is not injective on $W_{N-3}^{\partial ,(1,2)}$ (Lemma 5). The workaround is to separately consider the components of $d_{\omega } e$ in ${\mathcal {T}}$ and the components of $\omega$ in ${\mathcal {K}}$ (where ${\mathcal {T}}$ and ${\mathcal {K}}$ are introduced in (9)). Indeed, in the following theorem we consider a weaker version of the structural constraint (14) that generalizes it for a degenerate metric. This theorem is the generalization of Theorem 15.

#### Theorem 19

Let $g^{\partial }$ be degenerate. Given any ${\widetilde{\omega }} \in \Omega _{\partial }^{1,2}$, there is a unique decomposition

19 $$\begin{aligned} {\widetilde{\omega }}= \omega +v \end{aligned}$$

with $\omega$ and *v* satisfying

20a $$\begin{aligned}&e^{N-3}v=0, \end{aligned}$$

20b $$\begin{aligned}&e_n e^{N-4} d_{\omega } e - e_n e^{N-4} p_{{\mathcal {T}}}(d_{\omega } e) \in {{\,\mathrm{Im}\,}}W_{N-3}^{\partial ,(1,1)}, \end{aligned}$$

20c $$\begin{aligned}&p_{{\mathcal {K}}} v = 0. \end{aligned}$$

The proof is based on the following two lemmas generalizing, respectively, Lemmas 13 and 14.

#### Lemma 20

Let $g^{\partial }$ be degenerate, and let $\alpha \in \Omega ^{2,1}_\partial$. Then, $\alpha =0$ if and only if

21 $$\begin{aligned} {\left\{ \begin{array}{ll} e^{N-3}\alpha =0 \\ e_n e^{N-4}\alpha - e_n e^{N-4}p_{{\mathcal {T}}}\alpha \in {{\,\mathrm{Im}\,}}W_{N-3}^{\partial , (1,1)}\\ p_{{\mathcal {T}}}\alpha = 0 \end{array}\right. }. \end{aligned}$$

#### Proof

Trivial generalization of Lemma 13. $\square$

#### Lemma 21

Let $\beta \in \Omega ^{N-2,N-2}_\partial$. If $g^\partial$ is degenerate, there exist a unique $v \in \mathrm {Ker} W_{N-3}^{\partial , (1,2)}$, a unique $\gamma \in \Omega _{\partial }^{1,1}$ and a unique $\theta \in {\mathcal {T}}$ such that

$$\begin{aligned} \beta = e^{N-3} \gamma + e_n e^{N-4} [v, e] + e_n e^{N-4} \theta . \end{aligned}$$

#### Proof

By definition of ${\mathcal {T}}$, it is clear that for each element $\alpha \in \mathrm {Ker}W_{N-3}^{\partial (2,1)}$, it is possible to find $\theta \in {\mathcal {T}}$ and $v \in \mathrm {Ker} W_{N-3}^{\partial , (1,2)}$ such that $\alpha = [v,e] + \theta .$ From the proof of Lemma 14, we also know that each element $\beta \in \Omega ^{N-2,N-2}_\partial$ can be written as $\beta = e^{N-3} \gamma + e_n e^{N-4} \alpha$ for some $\alpha \in \mathrm {Ker}W_{N-3}^{\partial (2,1)}$. Combining these two results, we get the claim. $\square$

#### Proof of Theorem 19

Let ${\widetilde{\omega }} \in \Omega _\partial ^{1,2}$. From Lemma 21, we deduce that there exist $\sigma \in \Omega _\partial ^{1,1}$, $v \in \text {Ker} W_1^{\partial ,(1,2)}$ and $\theta \in {\mathcal {T}}$ such that

$$\begin{aligned} e_n e^{N-4} d_{{\widetilde{\omega }}} e = e^{N-3} \sigma + e_n e^{N-4}[v,e] + e_n e^{N-4} \theta . \end{aligned}$$

We define $\omega := {\widetilde{\omega }} - v$. Then, $\omega$ and *v* satisfy (19) and (20). $\square$

In contrast with the non-degenerate case, this theorem does not fix completely the freedom of $\omega \in [\omega ]$. Hence, we require the following additional equation:

22 $$\begin{aligned} p_{{\mathcal {K}}} \omega =0. \end{aligned}$$

Hence, (20b) and (22) fix uniquely the representative in the equivalence class.^19^

### Independence from the Choices

In this section, we explore the independence of the analysis from the choices that we have made in the construction. We prove it through the following general theorem.

#### Theorem 22

Let $(P, \varpi )$ be a presymplectic manifold with kernel distribution *K*, smooth leaf space $({\underline{P}}, {\underline{\varpi }})$ and canonical projection $\pi : P \rightarrow {\underline{P}}$. Let *Q* be a submanifold of *P* such that

$$\begin{aligned} \rho := \pi |_{Q} : Q \rightarrow {\underline{P}} \end{aligned}$$

is a diffeomorphism. Then, $(Q, \varpi |_{Q})$ is a symplectic manifold and $\rho$ is a symplectomorphism.

#### Proof

For every $x\in P$, we have that the exact sequence

$$\begin{aligned} 0 \rightarrow K_x \rightarrow T_x P \overset{d_x\pi }{\rightarrow } T_{\pi (x)} {\underline{P}} \rightarrow 0. \end{aligned}$$

For $x\in Q$, we have the splitting $d_{\rho (x)}: T_{\pi (x)}{\underline{P}}\rightarrow T_x P$ with image $T_x Q$ which gives $T_xM = T_x Q \oplus K_x$. Let now $v \in (T_xQ)^{\perp }$, then $\varpi _x(v,w)=0$ $\forall w \in T_xQ$. Furthermore, $\varpi _x(v,w)= \varpi _x (v, w + {\widetilde{w}})$ for all ${\widetilde{w}} \in K_x$. From the previous result, we get that $\varpi _x (v, {\widehat{w}})=0$ for all ${\widehat{w}} \in T_x P$. This implies that $v \in (T_x P)^{\perp }=K_x$. Therefore, $(T_xQ)^{\perp } \subseteq K_x$ and

$$\begin{aligned} (T_xQ)^{\perp } \cap T_xQ \subseteq K_x \cap T_xQ = \emptyset . \end{aligned}$$

Hence, $(Q, \varpi |_{Q})$ is symplectic.

From the definition of leaf space, we have that

$$\begin{aligned} \varpi _x(v,w) = {\underline{\varpi }}_{\pi (x)}([v],[w]) \qquad \forall x \in P \; \forall v,w \in T_x P \end{aligned}$$

Restricted to *Q*, this becomes

$$\begin{aligned} \varpi _x(v,w) = {\underline{\varpi }}_{\rho (x)}([v],[w]) \qquad \forall x \in Q \; \forall v,w \in T_x Q. \end{aligned}$$

Since $\rho$ is a diffeomorphism and $(Q, \varpi |_{Q})$ is a symplectic manifold, this last equation proves that $\rho$ is a symplectomorphism. $\square$

#### Corollary 23

If *Q* and $Q'$ are submanifolds of *P* such that $\pi |_{Q}$ and $\pi |_{Q'}$ are diffeomorphisms with ${\underline{P}}$, then $(Q, \varpi |_{Q})$ and $(Q', \varpi |_{Q'})$ are canonically symplectomorphic.

#### Remark 24

In our case, *P* is the space of restrictions to the boundary ${\widetilde{F}}_{PC}$ with presymplectic form ${\check{\varpi }}$, and *Q* is the subspace of ${\widetilde{F}}_{PC}$ where $\omega$ satisfies the constraints (20b) and (22), while ${\underline{P}}$ is the geometric phase space $F_{PC}^{\partial }$ with symplectic form $\varpi _{PC}^{\partial }$ defined in (12). The map $\pi$ is given by $\pi _{PC}$ defined in (11), and $\rho$ is its restriction to *Q*. The inverse of $\rho$ is given by the map $(e,[\omega ]) \mapsto (e, \omega ')$ where $\omega '$ is the unique representative of the class $[\omega ]$ satisfying (20b) and (22).

The existence of a canonical symplectomorphism between the constructions corresponding to different possible choices of the representative in the equivalence class of $[\omega ]$ guarantees the independence of the construction on such choices. In particular, the choice of the projection that leads to (22) is immaterial in the construction since we do not use this constraints anywhere else.

### Constraints of the Theory

Let us now turn to the constraints of the theory. In the degenerate case, we can still adopt the approach of the non-degenerate one adapting it to encompass the differences between Lemmas 13 and 20. The main difference is that now the constraint $L_c$ together with the new structural constraint (20b) is no longer equivalent to $d_{\omega }e=0$ (one set of the Euler–Lagrange equations in the bulk) since we are missing the third equation in (21). Indeed, we have to add an additional constraint that, thanks to Lemma 10, we can express as

23 $$\begin{aligned} R_{\tau } = \int _{\Sigma } \tau d_{\omega } e \end{aligned}$$

through an odd Lagrange multiplier $\tau \in {\mathcal {S}}[1]$.^20^ Furthermore, to simplify the computation of the brackets between the constraints, it is useful to modify the constraint $H_{\lambda }$ by adding to it a term proportional to $R_{\tau }:$

24 $$\begin{aligned} H_{\lambda } = \int _{\Sigma } \lambda e_n \left( \frac{1}{(N-3)}e^{N-3}F_\omega -e^{N-4}(\omega -\omega _0)p_{{\mathcal {T}}}(d_{\omega }e)+ \frac{1}{(N-1)!}\Lambda e^{N-1}\right) .\nonumber \\ \end{aligned}$$

Note that we can as well express the second term in this constraint as

$$\begin{aligned} \lambda p_{{\mathcal {S}}} ( e_n e^{N-4}(\omega -\omega _0))d_{\omega }e \end{aligned}$$

to make it explicitly in the form of (23).

#### Remark 25

The additional part in $H_{\lambda }$ proportional to $R_{\tau }$ has been added only to ease the computation of the Hamiltonian vector field of the constraint $H_{\lambda }$ itself. Such a linear combination does not affect the constrained set and the structure of the constraints, i.e. the distinction between first and second class constraints (see Proposition 35 and Remark 37 in “Appendix A”). Similar considerations hold also for the part of the constraint $P_{\xi }$ proportional to $L_c$, as already mentioned in [13, Remark 4.24] and [5, Remark 21].

Before analysing the structure of these constraints and their Poisson brackets, we need some additional results concerning the elements in ${\mathcal {S}}$ whose variations are constrained and are thus depending on *e*.

#### Lemma 26

The variation of an element $\tau \in {\mathcal {S}}$ is constrained by the following equations:

$$\begin{aligned} p_{{\widetilde{\rho }}}'\delta \tau&= {\widetilde{\rho }}^{-1}\left( \frac{\delta {\widetilde{\rho }}}{\delta e } (\tau ) \delta e\right) , \\ p_{W}'\delta \tau&= W_1^{-1}(\tau \delta e) \end{aligned}$$

where the inverses^21^ are defined on their images and $p_{{\widetilde{\rho }}}'$ and $p_{W}'$ are, respectively, the projections to a complement of the kernel of ${\widetilde{\rho }}$ and $W_1^{\partial , (N-3,N-1)}$.

#### Remark 27

Different choices of projections lead to different terms in the kernel of the two maps. Nonetheless, these additional terms are in ${\mathcal {S}}$ where the variation is free. Hence, they will not play any role in the computations.

#### Proof

From (9c), we know the elements $\tau \in {\mathcal {S}}$ must satisfy the following equations:

$$\begin{aligned} \tau \wedge e =0; \quad {\widetilde{\rho }}(\tau )=0. \end{aligned}$$

Hence, varying each equation we obtain some constraints for the variation $\delta \tau$:

$$\begin{aligned} \delta \tau \wedge e - \tau \wedge \delta e =0 ; \quad {\widetilde{\rho }}(\delta \tau ) + \frac{\delta {\widetilde{\rho }}}{\delta e } (\tau ) \delta e =0. \end{aligned}$$

We can invert these equations using the inverses of $W_1^{\partial , (N-3, N-1)}$ and ${\widetilde{\rho }}$ on their images. Denoting with $p'_W$ and $p'_{{\widetilde{\rho }}}$ the projections to some complements of the kernel of $W_1^{\partial , (N-3, N-1)}$ and ${\widetilde{\rho }}$ in $\Omega _{\partial }^{N-3, N-1}$, respectively, we obtain

$$\begin{aligned} p'_W \delta \tau = W_1^{-1} (\tau \wedge \delta e ) ; \quad p'_{{\widetilde{\rho }}} \delta \tau = {\widetilde{\rho }}^{-1}\left( \frac{\delta {\widetilde{\rho }}}{\delta e } (\tau ) \delta e\right) . \end{aligned}$$

These relations fix the constrained part of the variation of $\tau \in {\mathcal {S}}$ in terms of the variation of *e*. $\square$

#### Lemma 28

The following identities hold:

$$\begin{aligned} {\widetilde{\rho }}^{-1}\left( \frac{\delta {\widetilde{\rho }}}{\delta e } (\tau ) [c,e]\right) =p'_{{\widetilde{\rho }}}[c, \tau ], \qquad {\widetilde{\rho }}^{-1}\left( \frac{\delta {\widetilde{\rho }}}{\delta e } (\tau ) {\mathcal {L}}_{\xi }^{\omega _0} e\right) p'_{{\widetilde{\rho }}}{\mathcal {L}}_{\xi }^{\omega _0}\tau . \end{aligned}$$

#### Proof

We start by making more explicit the expression ${\widetilde{\rho }}^{-1}\left( \frac{\delta {\widetilde{\rho }}}{\delta e } (\tau ) \delta e\right)$. By definition, if $\tau \in {\mathcal {S}}$, then $[\tau , {\widetilde{e}}]=0$. Hence,

$$\begin{aligned} 0 = \delta [\tau , {\widetilde{e}}] = [\delta \tau , {\widetilde{e}}] + [\tau , \delta {\widetilde{e}}]. \end{aligned}$$

We now compute $\delta {\widetilde{e}}$ in terms of $\delta e$:

$$\begin{aligned} \delta {\widetilde{e}}= \delta e - \delta {\widehat{e}}= \delta e - \delta (\beta \iota _X e)= \delta e - \delta \beta \iota _X e + \beta \iota _{\delta X} e - \beta \iota _X \delta e. \end{aligned}$$

We have then to compute the variation $\delta X$ and $\delta \beta$. We start from the first: from the defining equation $\iota _X g^{\partial }=0$, we get

$$\begin{aligned} \iota _{\delta X} g^{\partial } - \iota _X \delta g^{\partial }=0 \end{aligned}$$

and hence, inverting $g^{\partial }$ on its image, we get $\delta X= {g^{\partial }}^{-1}(\iota _X \delta g^{\partial })$. Since $g^{\partial }$ can be written in terms of *e* and $\eta$ as $g^{\partial }= \eta (e,e)$, we can write this part of $\delta X$ in terms of $\delta e$. The remaining part of $\delta X$ not fixed by this equation is such that $\iota _{\delta X} g^{\partial }=0$, and hence,

$$\begin{aligned} \delta X= 2{g^{\partial }}^{-1}(\iota _X \eta ( \delta e , e )) + \lambda X \end{aligned}$$

for some function $\lambda$.

Let us now pass to $\delta \beta$. Its value is completely determined by the equations $\iota _{X}\delta \beta - \iota _{\delta X} \beta =0$ and

$$\begin{aligned} \iota _{Y_0^1}\dots \iota _{Y_0^{N-2}}&\left( \iota _{\delta X} (\beta e^{N-3}) v - \iota _X(\delta \beta e^{N-3}) v \right) \\&+ \iota _{Y_0^1}\dots \iota _{Y_0^{N-2}}\left( (N-3)\iota _X(\beta \delta e e^{N-4}) v + \iota _X (\beta e^{N-3}) \delta v\right) = 0. \end{aligned}$$

This last equation must hold for every *v* and $\delta v$ that satisfy, respectively, $e^{N-3} \wedge v = 0$ and $(N-3) \delta e e^{N-4} v + e^{N-3}\delta v=0$.

We can now plug the values $\delta e= [c,e]$ and $\delta e= {\mathcal {L}}_{\xi }^{\omega _0} e$ in the first formula of Lemma 26 using the above results. In the first case, we get

$$\begin{aligned} \delta X= 2{g^{\partial }}^{-1}(\iota _X [[c,e] , e ])+ \lambda X= 2{g^{\partial }}^{-1}(\iota _X [c,[e , e ]])+ \lambda X= \lambda X \end{aligned}$$

and $$ \delta \beta = \lambda \beta $$. Consequently,

$$\begin{aligned} {\widetilde{\rho }}^{-1}\left( [\tau , [c,e] - \beta \iota _X [c,e]] \right)&={\widetilde{\rho }}^{-1}\left( [\tau , [c,e] - [c, \beta \iota _X e]] \right) \\&= {\widetilde{\rho }}^{-1}\left( [\tau , [c,{\widetilde{e}}] \right) \\&= {\widetilde{\rho }}^{-1}\left( [[\tau , c],{\widetilde{e}}] +[c, [\tau , {\widetilde{e}}]]\right) \\&= p'_{{\widetilde{\rho }}} [\tau , c]. \end{aligned}$$

In the second case, we have

$$\begin{aligned} \delta X= 2{g^{\partial }}^{-1}(\iota _X [{\mathcal {L}}_{\xi }^{\omega _0} e , e ])+ \lambda X= {g^{\partial }}^{-1}(\iota _X {\mathcal {L}}_{\xi }^{\omega _0} g^{\partial })+ \lambda X. \end{aligned}$$

and $\delta \beta = {\mathcal {L}}_{\xi }^{\omega _0} \beta + \lambda \beta$. In coordinates we obtain the following expressions

$$\begin{aligned} \delta X^{\mu }&= X^{\rho } \partial _{\rho } \xi ^{\mu } + \xi ^{\rho } \partial _{\rho } X^{\mu } + \lambda X^{\mu }\\ \iota _X {\mathcal {L}}_{\xi }^{\omega _0} e&= X^{\rho } \xi ^{\mu }{ d_{\omega _0}}_{\mu } e_{\rho } - X^{\rho } e_{\mu } d_{\rho } \xi ^{\mu }. \end{aligned}$$

Hence

$$\begin{aligned} \iota _X {\mathcal {L}}_{\xi }^{\omega _0} e + \iota _{\delta X} e= \iota _{\xi } d_{\omega } (\iota _X e )+ \lambda \iota _X e , \end{aligned}$$

and collecting all these formulas, we get

$$\begin{aligned} {\widetilde{\rho }}^{-1}\left( \frac{\delta {\widetilde{\rho }}}{\delta e } (\tau ) {\mathcal {L}}_{\xi }^{\omega _0} e\right)&= {\widetilde{\rho }}^{-1}\left( [\tau ,{\mathcal {L}}_{\xi }^{\omega _0} e - {\mathcal {L}}_{\xi }^{\omega _0} (\beta \iota _X e )]\right) \\&= {\widetilde{\rho }}^{-1}\left( [\tau ,{\mathcal {L}}_{\xi }^{\omega _0} {\widetilde{e}}] \right) \\&= {\widetilde{\rho }}^{-1}\left( {\mathcal {L}}_{\xi }^{\omega _0}[\tau , {\widetilde{e}}] - [{\mathcal {L}}_{\xi }^{\omega _0}\tau , {\widetilde{e}}]\right) \\&= p'_{{\widetilde{\rho }}}{\mathcal {L}}_{\xi }^{\omega _0}\tau . \end{aligned}$$

$$\square $$

The addition of the constraint $R_{\tau }$ to compensate the different structure of the lightlike case has important consequences on the structure of the set of constraints.

#### Theorem 29

Let $g^\partial$ be degenerate on $\Sigma$. Then, the structure of the Poisson brackets of the constraints $L_c$, $P_{\xi }$, $H_{\lambda }$ and $R_{\tau }$ is given by the following expressions:

$$\begin{aligned}&\begin{aligned}&\{L_c, L_c\} = - \frac{1}{2} L_{[c,c]}&\qquad \qquad&\{P_{\xi }, P_{\xi }\} = \frac{1}{2}P_{[\xi , \xi ]}- \frac{1}{2}L_{\iota _{\xi }\iota _{\xi }F_{\omega _0}} \\&\{L_c, P_{\xi }\} = L_{{\mathcal {L}}_{\xi }^{\omega _0}c}&\{H_{\lambda },H_{\lambda }\} \approx F_{\tau ' \tau '} \\&\{L_c, R_{\tau }\} = -R_{p_{{\mathcal {S}}}[c, \tau ]}&\{P_{\xi },R_{\tau }\} = R_{p_{{\mathcal {S}}}{\mathcal {L}}_{\xi }^{\omega _0}\tau }.\\&\{ R_{\tau }, H_{\lambda } \} \approx F_{\tau \tau '} + G_{\lambda \tau }&\{R_{\tau },R_{\tau }\} \approx F_{\tau \tau } \end{aligned}\\&\{L_c, H_{\lambda }\} = - P_{X^{(a)}} + L_{X^{(a)}(\omega - \omega _0)_a} - H_{X^{(n)}} + R_{p_{{\mathcal {S}}}(X^{(a)}e_a e^{N-4} (\omega - \omega _0)-\lambda e_n d_{\omega _0} c)} \\&\{P_{\xi },H_{\lambda }\} = P_{Y^{(a)}} -L_{ Y^{(a)} (\omega - \omega _0)_a} +H_{ Y^{(n)}}-R_{p_{{\mathcal {S}}}(Y^{(a)} e_a e^{N-4} (\omega - \omega _0) -\lambda e_n \iota _ {\xi }F_{\omega _0})} \end{aligned}$$

where $\tau '= p_{{\mathcal {S}}}(\lambda e_n e^{N-4} (\omega -\omega _0))$, $X= [c, \lambda e_n ]$, $Y = {\mathcal {L}}_{\xi }^{\omega _0} (\lambda e_n)$ and $Z^{(a)}$, $Z^{(n)}$ are the components of $Z\in \{X,Y\}$ with respect to the frame $(e_a, e_n)$. Furthermore, $F_{\tau \tau }$, $F_{\tau \tau '}$, $F_{\tau ' \tau '}$ and $G_{\lambda \tau }$ are functions of *e*, $\omega$, $\tau$ (or $\tau '$) and $\lambda$ defined in the proof that are not proportional to any other constraint.

#### Remark 30

In Theorem 29, we use the symbol $\approx$ to denote the fact that the result can be obtained only working on shell, i.e. imposing the constraints. Here, we want to stress that the brackets are not proportional to the constraints, while in the other cases (the ones with the $=$ sign), we get an exact result. Equivalently, we could have written, e.g. $\{L_c, L_c\} \approx 0$.

#### Proof

We first compute the variation of the constraints in order to find their Hamiltonian vector fields. Using the results of [5] for $L_c$ and $P_{\xi }$, we have:

$$\begin{aligned}&\delta L_c= \int _{\Sigma } -\frac{1}{N-2} c [\delta \omega , e^{N-2}] + \frac{1}{N-2} c d_{\omega }\delta (e^{N-2}) \\&\quad = \int _{\Sigma } [c, e] e^{N-3} \delta \omega + d_{\omega } c e^{N-3}\delta e; \\&\delta P_{\xi } = \int _{\Sigma } \iota _{\xi } (e^{N-3} \delta e) F_{\omega } -\frac{1}{N-2}\iota _{\xi } (e^{N-2}) d_{\omega }\delta \omega + \iota _{\xi } \delta \omega e^{N-3} d_{\omega } e \\&\qquad -\frac{1}{N-2} \iota _{\xi } (\omega -\omega _0) [\delta \omega , e^{N-2}] + \frac{1}{N-2} \iota _{\xi } (\omega -\omega _0) d_{\omega }\delta (e^{N-2}) \\&\quad = \int _{\Sigma } - e^{N-3} \delta e ({\mathcal {L}}_{\xi }^{\omega _0} (\omega -\omega _0) + \iota _ {\xi }F_{\omega _0}) - ({\mathcal {L}}_{\xi }^{\omega _0} e ) e^{N-3} \delta \omega ;\\&\delta R_{\tau } = \int _{\Sigma } \delta _e \tau d_{\omega }e - \tau [\delta \omega ,e] + \tau d_{\omega } \delta e \\&\quad \; = \int _{\Sigma } \delta e g(\tau ,\omega ,e) + [\tau , e] \delta \omega + d_{\omega } \tau \delta e \end{aligned}$$

where $g(\tau ,\omega ,e)$ is a formal expression that encodes the dependence of $\delta \tau$ on $\delta e$, i.e. such that

$$\begin{aligned} \delta e g(\tau ,\omega ,e)&= p'_{{\widetilde{\rho }}} {\widetilde{\rho }}^{-1}\left( \frac{\delta {\widetilde{\rho }}}{\delta e } (\tau ) \delta e\right) d_{\omega }e + p'_{W} W_1^{-1}(\tau \delta e)d_{\omega }e\\ {}&\quad - p'_{X} {\widetilde{\rho }}^{-1}\left( \frac{\delta {\widetilde{\rho }}}{\delta e } (\tau ) \delta e\right) d_{\omega }e \end{aligned}$$

as shown in Lemma 26 where $p'_{X}$ is the projection to the intersection of the complement of the kernel of ${\widetilde{\rho }}$ and $W_1^{\partial , (N-3,N-1)}$. Using this last computation, we can compute the variation of the Hamiltonian constraint $H_{\lambda }$:

$$\begin{aligned} \delta H_{\lambda }&= \int _{\Sigma } \lambda e_n e^{N-4}\delta e F_{\omega }+\frac{1}{(N-2)!}\Lambda \lambda e_n e^{N-2} \delta e -\frac{1}{(N-3)}\lambda e_n e^{N-3} d_{\omega } \delta \omega \\&\quad - \lambda p_{{\mathcal {S}}} (e_n e^{N-4}\delta \omega ) d_{\omega }e - (N-4)\lambda p_{{\mathcal {S}}} (e_n e^{N-5}\delta e (\omega -\omega _0)) d_{\omega }e \\&\quad - \delta _e \tau ' d_{\omega }e + \tau ' [\delta \omega , e] -\tau ' d_{\omega }\delta e\\&= \int _{\Sigma } \lambda e_n e^{N-4}\delta e F_{\omega } +\frac{1}{(N-2)!}\Lambda \lambda e_n e^{N-2} \delta e+ \frac{1}{(N-3)} d_{\omega }(\lambda e_n) e^{N-3} \delta \omega \\&\quad + \lambda e_n e^{N-4}d_{\omega } e \delta \omega - \lambda e_n e^{N-4} \delta \omega p_{{\mathcal {T}}}(d_{\omega }e)\\&\quad - (N-4)\lambda e_n e^{N-5}\delta e (\omega -\omega _0) p_{{\mathcal {T}}}(d_{\omega }e) - \delta e g(\tau ',\omega ,e)\\ {}&\quad + \tau ' [\delta \omega , e] -\tau ' d_{\omega }\delta e\\&= \int _{\Sigma } \lambda e_n e^{N-4}\delta e F_{\omega } +\frac{1}{(N-2)!}\Lambda \lambda e_n e^{N-2} \delta e+ \frac{1}{(N-3)} d_{\omega }(\lambda e_n) e^{N-3} \delta \omega \\&\quad + \lambda \sigma e^{N-3} \delta \omega - (N-4)\lambda e_n e^{N-5}\delta e (\omega -\omega _0) p_{{\mathcal {T}}}(d_{\omega }e) \\&\quad - \delta e g(\tau ',\omega ,e) + \tau ' [\delta \omega , e] -\tau ' d_{\omega }\delta e \end{aligned}$$

where $\tau '= p_{{\mathcal {S}}}(\lambda e_n e^{N-4} (\omega -\omega _0))$ and we used (20b). From the expressions of the variation of the constraints, we can deduce their Hamiltonian vector fields. Let *X* be a generic constraint, then we denote with ${\mathbb {X}}$ the corresponding Hamiltonian vector field $\iota _{{\mathbb {X}}}\varpi ^{\partial }_{PC}= \delta X$ and with ${\mathbb {X}}_e$ ${\mathbb {X}}_{\omega }$ its components, i.e.

$$\begin{aligned} {\mathbb {X}} = {\mathbb {X}}_e \frac{\delta }{\delta e} + {\mathbb {X}}_{\omega } \frac{\delta }{\delta \omega }. \end{aligned}$$

Hence we have

$$\begin{aligned}&\begin{aligned}&{\mathbb {L}}_e = [c,e]&\qquad \qquad \qquad \qquad&{\mathbb {L}}_\omega = d_{\omega } c \\&{\mathbb {P}}_e = - {\mathcal {L}}_{\xi }^{\omega _0} e&{\mathbb {P}}_\omega = - {\mathcal {L}}_{\xi }^{\omega _0} (\omega -\omega _0) - \iota _ {\xi }F_{\omega _0}\\&e^{N-3} {\mathbb {R}}_e= [\tau , e]&e^{N-3} {\mathbb {R}}_\omega = g(\tau ,\omega ,e) + d_{\omega } \tau \end{aligned}\\&e^{N-3} {\mathbb {H}}_e = \frac{1}{(N-3)} e^{N-3} d_{\omega }(\lambda e_n) + \lambda e^{N-3} \sigma - [\tau ', e]\\&e^{N-3} {\mathbb {H}}_\omega = \lambda e_n e^{N-4} F_{\omega }+\frac{1}{(N-2)!}\Lambda \lambda e_n e^{N-2}\\&\qquad \qquad - (N-4)\lambda e_n e^{N-5}(\omega -\omega _0)p_{{\mathcal {T}}}(d_{\omega }e)- g(\tau ',\omega ,e) - d_{\omega } \tau '. \end{aligned}$$

The components ${\mathbb {R}}_\omega$ and ${\mathbb {H}}_\omega$ are uniquely determined requiring the structural constraint (20b). The components ${\mathbb {R}}_e$ and ${\mathbb {H}}_e$ are recovered by inversion of $W_{N-3}^{\partial , (1,1)}$ (which is possible thanks to Lemma 11). Following these, we compute the Poisson brackets between the constraints and analyse their structure. The brackets between $L_c$ and $P_{\xi }$ are the same as in the non-degenerate case presented in [5]:

$$\begin{aligned} \{L_c, L_c\} = - \frac{1}{2} L_{[c,c]}; \quad \{L_c, P_{\xi }\} = L_{{\mathcal {L}}_{\xi }^{\omega _0}c}; \quad \{P_{\xi }, P_{\xi }\} = \frac{1}{2}P_{[\xi , \xi ]} - \frac{1}{2}L_{\iota _{\xi }\iota _{\xi }F_{\omega _0}}. \end{aligned}$$

Let us now compute the brackets between $L_c$, $P_{\xi }$ and $R_{\tau }$. In both computations, we use the results of Lemmas 26 and 28 and the properties of $\tau$.

$$\begin{aligned} \{L_c, R_{\tau }\}= \int _{\Sigma }&[c,e] g(\tau ,\omega ,e) + [c,e]d_{\omega } \tau + d_{\omega } c [\tau , e] \\ = \int _{\Sigma }&[c,e] g(\tau ,\omega ,e)- [c, \tau ] d_{\omega } e \\ = \int _{\Sigma }&p'_{{\mathcal {S}}}[c, \tau ] d_{\omega } e- [c, \tau ] d_{\omega } e = \int _{\Sigma } - p_{{\mathcal {S}}}[c, \tau ] d_{\omega } e = -R_{p_{{\mathcal {S}}}[c, \tau ]}; \\ \{P_{\xi },R_{\tau }\}= \int _{\Sigma }&- [\tau , e]{\mathcal {L}}_{\xi }^{\omega _0} (\omega -\omega _0) - [\tau , e]\iota _ {\xi }F_{\omega _0} - {\mathcal {L}}_{\xi }^{\omega _0} e g(\tau ,\omega ,e) - {\mathcal {L}}_{\xi }^{\omega _0} e d_{\omega } \tau \\ = \int _{\Sigma }&- {\mathcal {L}}_{\xi }^{\omega _0} e g(\tau ,\omega ,e) + {\mathcal {L}}_{\xi }^{\omega _0}\tau d_{\omega } e\\ = \int _{\Sigma }&-p'_{{\mathcal {S}}}{\mathcal {L}}_{\xi }^{\omega _0}\tau d_{\omega } e+ {\mathcal {L}}_{\xi }^{\omega _0}\tau d_{\omega } e = \int _{\Sigma } p_{{\mathcal {S}}}{\mathcal {L}}_{\xi }^{\omega _0}\tau d_{\omega } e = R_{p_{{\mathcal {S}}}{\mathcal {L}}_{\xi }^{\omega _0}\tau }. \end{aligned}$$

We now compute the brackets between $L_c$, $P_{\xi }$ and $H_{\lambda }$.

$$\begin{aligned} \{L_c, H_{\lambda }\}&= \int _{\Sigma } [c,e] e^{N-4} \lambda e_n F_{\omega } +\frac{1}{(N-2)!}[c,e]\Lambda \lambda e_n e^{N-2} -[c,e]g(\tau ',\omega ,e) \\&\quad -[c,e]d_{\omega } \tau ' - (N-4) [c,e] \lambda e_n e^{N-5}(\omega -\omega _0)p_{{\mathcal {T}}}(d_{\omega }e) \\&\quad + \frac{1}{(N-3)} e^{N-3} d_{\omega } c d_{\omega }(\lambda e_n) + e^{N-3} d_{\omega } c\lambda \sigma - d_{\omega } c [\tau ', e]\\&= \int _{\Sigma } - \frac{1}{(N-3)} [c, \lambda e_n ] e^{N-3} F_{\omega }-\frac{1}{(N-1)!}\Lambda [c, \lambda e_n ] e^{N-1} \\&\quad + p_{{\mathcal {S}}}([c, \tau '] - \lambda e_n e^{N-4}d_{\omega } c - [c,e^{N-4}] \lambda e_n (\omega -\omega _0))d_{\omega }e\\&= \int _{\Sigma } - \frac{1}{(N-3)}\left( [c, \lambda e_n ]^{(a)}e_a e^{N-3} F_{\omega } -[c, \lambda e_n ]^{(n)}e_n e^{N-3} F_{\omega }\right) \\&\quad -\frac{1}{(N-1)!}\Lambda [c, \lambda e_n ]^{(n)}e_n e^{N-1} - p_{{\mathcal {S}}}(\lambda e_n e^{N-4} d_{\omega _0} c ) d_{\omega } e\\&\quad +p_{{\mathcal {S}}}([c, \lambda e_n ]^{(a)}e_a e^{N-4}(\omega - \omega _0) +[c, \lambda e_n ]^{(n)}e_n e^{N-4}(\omega - \omega _0) ) d_{\omega } e \\&= - P_{[c, \lambda e_n ]^{(a)}} + L_{[c, \lambda e_n ]^{(a)}(\omega - \omega _0)_a} - H_{[c, \lambda e_n ]^{(n)}}\\&\quad + R_{p_{{\mathcal {S}}}([c, \lambda e_n ]^{(a)}e_a e^{N-4}(\omega - \omega _0))} - R_{p_{{\mathcal {S}}}(\lambda e_n e^{N-4}d_{\omega _0} c)}; \\&\{P_{\xi },H_{\lambda }\} = \int _{\Sigma } - {\mathcal {L}}_{\xi }^{\omega _0} e \lambda e_n e^{N-4}F_{\omega } \\&\quad -\frac{1}{(N-2)!}\Lambda {\mathcal {L}}_{\xi }^{\omega _0} e \lambda e_n e^{N-2}+ {\mathcal {L}}_{\xi }^{\omega _0} e g(\tau ',\omega ,e)\\&\quad + {\mathcal {L}}_{\xi }^{\omega _0} e d_{\omega } \tau '+ (N-4) {\mathcal {L}}_{\xi }^{\omega _0} e \lambda e_n e^{N-5}(\omega -\omega _0)p_{{\mathcal {T}}}(d_{\omega }e)\\&\quad - \left( {\mathcal {L}}_{\xi }^{\omega _0} (\omega -\omega _0)+ \iota _ {\xi }F_{\omega _0}\right) \left( \frac{e^{N-3} d_{\omega }(\lambda e_n)}{N-3} + \lambda e^{N-3} \sigma - [\tau ', e] \right) \\&= \int _{\Sigma } \frac{1}{(N-3)} {\mathcal {L}}_{\xi }^{\omega _0} (\lambda e_n) e^{N-3} F_{\omega } +\frac{1}{(N-1)!}\Lambda e^{N-1} {\mathcal {L}}_{\xi }^{\omega _0}(\lambda e_n) \\&\quad + p_{{\mathcal {S}}}\left( -{\mathcal {L}}_{\xi }^{\omega _0} \tau '+ \lambda e_n e^{N-4}\left( {\mathcal {L}}_{\xi }^{\omega _0} (\omega -\omega _0)+ \iota _ {\xi }F_{\omega _0}\right) \right) d_{\omega } e \\&\quad + p_{{\mathcal {S}}}({\mathcal {L}}_{\xi }^{\omega _0}( e^{N-4}) \lambda e_n (\omega -\omega _0))d_{\omega }e\\&= \int _{\Sigma } \frac{1}{(N-3)} \left( {\mathcal {L}}_{\xi }^{\omega _0} (\lambda e_n)^{(a)}e_a e^{N-3} F_{\omega }+{\mathcal {L}}_{\xi }^{\omega _0} (\lambda e_n)^{(n)}e_n e^{N-3} F_{\omega } \right) \\&\quad +\frac{1}{(N-1)!}\Lambda e^{N-1} {\mathcal {L}}_{\xi }^{\omega _0}(\lambda e_n)^{(n)}e_n + p_{{\mathcal {S}}} (\lambda e_n e^{N-4}\iota _ {\xi }F_{\omega _0})d_{\omega } e\\&\quad - p_{{\mathcal {S}}}\left( {\mathcal {L}}_{\xi }^{\omega _0}(\lambda e_n)^{(n)} e_n e^{N-4}(\omega - \omega _0) \right. \\&\quad \left. + {\mathcal {L}}_{\xi }^{\omega _0}(\lambda e_n)^{(a)} e_a e^{N-4}(\omega - \omega _0) \right) d_{\omega } e \\&= P_{ {\mathcal {L}}_{\xi }^{\omega _0} (\lambda e_n)^{(a)}} +H_{ {\mathcal {L}}_{\xi }^{\omega _0} (\lambda e_n)^{(n)}}-L_{ {\mathcal {L}}_{\xi }^{\omega _0} (\lambda e_n)^{(a)} (\omega - \omega _0)_a}\\&\quad -R_{p_{{\mathcal {S}}}({\mathcal {L}}_{\xi }^{\omega _0}(\lambda e_n)^{(a)} e_a e^{N-4}(\omega - \omega _0))} + R_{p_{{\mathcal {S}}}(\lambda e_n e^{N-4}\iota _ {\xi }F_{\omega _0})}. \end{aligned}$$

We now compute the remaining brackets $\{R_{\tau },R_{\tau }\}$, $\{R_{\tau },H_{\lambda }\}$ and $\{H_{\lambda },H_{\lambda }\}$. Since $H_{\lambda }$ contains terms proportional to $R_{\tau }$ (for $\tau = p_{{\mathcal {S}}}( \lambda e_n e^{N-4} (\omega - \omega _0)$)), we first compute the brackets between two $R_{\tau }$ and then the others:

$$\begin{aligned} \{R_{\tau },R_{\tau }\}= \int _{\Sigma }&W_{N-3}^{-1}([\tau , e])g(\tau ,\omega ,e) + W_{N-3}^{-1}([\tau , e]) d_{\omega } \tau . \end{aligned}$$

The first term is proportional to $d_{\omega }e$ by construction, so it will be 0 on shell. Let us concentrate on the second term. We want to prove, using normal geodesic coordinates, that it is not proportional to any of the constraints and not 0. Let us fix a point $p \in \Sigma$ and consider an open neighbourhood *U* of it. From Proposition 8, we deduce that the unique components at the point *p* with respect to the standard basis that compose $\tau$ are $X_{\mu _2}^{\mu _1}, Y_{\mu }$ for $\mu , \mu _1, \mu _2=1 \dots N-2$ subject to

$$\begin{aligned} \sum _{\mu =1}^{N-2} Y_{\mu } =0 \text { and } X_{\mu _1}^{\mu _2} =- X_{\mu _2}^{\mu _1}. \end{aligned}$$

The first equation holds also on the whole neighbourhood, while the second set holds only on the point *p*. From Corollary 12, we know that the nonzero components in $W_{N-3}^{-1} ([\tau , e])$ are

$$\begin{aligned}{}[W_{N-3}^{-1}([\tau ,e])]_{\mu _1}^{\mu _2}&\propto X_{\mu _1}^{\mu _2} \\ [W_{N-3}^{-1}([\tau ,e])]_{\mu }^{\mu }&\propto Y_{\mu } \\ \end{aligned}$$

such that $\sum _{\mu =1}^{N-2} [W_{N-3}^{-1}([\tau ,e])]_{\mu }^{\mu }=0$ and $[W_{N-3}^{-1}([\tau ,e])]_{\mu _1}^{\mu _2}=-[W_{N-3}^{-1}([\tau ,e])]_{\mu _2}^{\mu _1}$.

Furthermore, from Proposition 8 we also know that the nonzero components of $\tau$ are $Y_{\mu }$ and $X_{\mu _1}^{\mu _2}$ such that

$$\begin{aligned} \sum _{\mu =1}^{N-2} Y_{\mu } =0 \text { and } X_{\mu _1}^{\mu _2} = f({\widetilde{g}}^{\partial }, X_{\mu _2}^{\mu _1}, Y_{\mu }) \end{aligned}$$

for $\mu _1 < \mu _2$ and some linear function *f*. Remembering that $W_{N-3}^{-1}([\tau , e]) d_{\omega } \tau$ should be a volume form, we deduce that, on shell,

$$\begin{aligned} W_{N-3}^{-1}([\tau , e]) d_{\omega } \tau&= \left( \sum _{\mu =1}^{N-2} Y_{\mu } \partial _{N-1} Y_{\mu } + \sum _{\mu _1, \mu _2=1}^{N-2} X_{\mu _1}^{\mu _2}\partial _{N-1} X_{\mu _2}^{\mu _1} \right) {\mathbf {V}} \\&= \left( \sum _{\mu =1}^{N-2} Y_{\mu } \partial _{N-1} Y_{\mu } +\!\! \sum _{\mu _1 < \mu _2 \mu _1,\mu _2=1}^{N-2}\! X_{\mu _1}^{\mu _2}\partial _{N-1} f({\widetilde{g}}^{\partial }, X_{\mu _1}^{\mu _2}, Y_{\mu }) \!\right) {\mathbf {V}}\\&= :F_{\tau \tau } \end{aligned}$$

where ${\mathbf {V}}= e_{1} \dots e_{N-1} e_n dx^{1} \dots dx^{N-1}$. This quantity is for generic $\tau$ different from zero, on shell. Hence,

$$\begin{aligned} \{ R_{\tau }, R_{\tau } \} \approx F_{\tau \tau } \not \approx 0. \end{aligned}$$

With this result, we can more easily compute the last two brackets:

$$\begin{aligned} \{H_{\lambda }, H_{\lambda }\}&= \int _{\Sigma } \left( \frac{1}{(N-3)} d_{\omega }(\lambda e_n)+\lambda \sigma - W_{N-3}^{-1}([\tau ', e])\right) \lambda e_n e^{N-4} F_{\omega }\\&\quad + \left( \frac{1}{(N-3)} d_{\omega }(\lambda e_n)+\lambda \sigma - W_{N-3}^{-1}([\tau ', e])\right) \frac{1}{(N-2)!}\Lambda \lambda e_n e^{N-2} \\&\quad - \left( \frac{1}{(N-3)} d_{\omega }(\lambda e_n)+\lambda \sigma - W_{N-3}^{-1}([\tau ', e])\right) g(\tau ',\omega ,e) \\&\quad - \left( \frac{1}{(N-3)} d_{\omega }(\lambda e_n)+\lambda \sigma - W_{N-3}^{-1}([\tau ', e])\right) d_{\omega } \tau '\\&\quad - (N-4)\frac{1}{(N-3)} d_{\omega }(\lambda e_n)\lambda e_n e^{N-5}(\omega -\omega _0)p_{{\mathcal {T}}}(d_{\omega }e)\\&\quad - (N-4) \left( \lambda \sigma - W_{N-3}^{-1}([\tau ', e])\right) \lambda e_n e^{N-5}(\omega -\omega _0)p_{{\mathcal {T}}}(d_{\omega }e). \end{aligned}$$

Since $\lambda$ and $e_n$ are odd quantities and $\tau '= \lambda p_{{\mathcal {S}}}(e_n e^{N-4}(\omega -\omega _0))$, the terms in the first two lines and in the last two vanish. Furthermore, the last terms of the third and fourth lines are the one composing the brackets $\{R_{\tau '},R_{\tau '}\}$. Expanding the first and the second term of the third line, we get

$$\begin{aligned}&{\widetilde{\rho }}^{-1}([\tau ', d_{\omega }(\lambda e_n)])d_{\omega }e + W_1^{-1}(\tau ' d_{\omega }(\lambda e_n))d_{\omega }e + {\widetilde{\rho }}^{-1}([\tau ', \lambda \sigma ])d_{\omega }e\\ {}&\quad + W_1^{-1}(\tau ' \lambda \sigma )d_{\omega }e. \end{aligned}$$

All these terms are zero since they encompass terms with either $$\lambda \lambda =0$$ or $$ e_n e_n=0$$. We can draw the same conclusion also for the following term:

$$\begin{aligned} d_{\omega }(\lambda e_n)d_{\omega } \tau '= [ F_{\omega }, \lambda e_n] \tau '=0. \end{aligned}$$

The same holds also for the term $\lambda \sigma d_{\omega } \tau '$ since both $\sigma$ and $\tau '$ contain $e_n$.^22^ Hence,

$$\begin{aligned} \{H_{\lambda }, H_{\lambda }\}=\{R_{\tau '},R_{\tau '}\} \approx F_{\tau ' \tau '} \not \approx 0. \end{aligned}$$

The last bracket that we have to compute is $\{ R_{\tau }, H_{\lambda } \}$. From the expression of the Hamiltonian vector fields, we get

$$\begin{aligned} \{ R_{\tau }, H_{\lambda } \}&= \int _{\Sigma } \frac{1}{(N-3)} d_{\omega }(\lambda e_n) \left( g(\tau ,\omega ,e)+d_{\omega } \tau \right) + \lambda \sigma g(\tau ,\omega ,e)+ \lambda \sigma d_{\omega } \tau \\&\quad + W_{N-3}^{-1}([\tau , e])\lambda e_n e^{N-4} F_{\omega }- W_{N-3}^{-1}([\tau ', e])\left( g(\tau ,\omega ,e)+d_{\omega } \tau \right) \\&\quad + \frac{1}{(N-2)!}\Lambda \lambda e_n e[\tau , e] - W_{N-3}^{-1}([\tau , e])\left( g(\tau ',\omega ,e)+d_{\omega } \tau ' \right) \\&\quad - (N-4)W_{N-3}^{-1}([\tau , e])\lambda e_n e^{N-5}(\omega -\omega _0)p_{{\mathcal {T}}}(d_{\omega }e). \end{aligned}$$

The last two terms of the second and third lines are the one composing the brackets $\{R_{\tau },R_{\tau '}\}$, and the first term of the third line vanishes because $e \tau =0$ and $[e,e]=0$. We want to prove that $\{ R_{\tau }, H_{\lambda } \} \not \approx 0$. Using coordinate expansion, one can prove that the second and the fifth terms have the same expression and read:

$$\begin{aligned} d_{\omega }(\lambda e_n)d_{\omega }\tau&+ W_1^{-1}([\tau , e])\lambda e_nF_{\omega } = - [F_{\omega } , \lambda e_n] \tau + W_1^{-1}([\tau , e])\lambda e_nF_{\omega } \\&=2 \lambda \sum _{\mu =1}^{N-2} Y_{\mu } (F_{\omega })_{\mu N-1 }^{\mu N-1} + \lambda \sum _{\mu _1, \mu _2=1}^{N-2} X_{\mu _1}^{\mu _2}(F_{\omega })_{\mu _2 N-1 }^{\mu _1 N-1} = :G_{\lambda \tau }. \end{aligned}$$

These terms are not proportional to any of the constraints and not proportional to $\{R_{\tau },R_{\tau '}\}$. The term in the fourth line is proportional to $R_{\tau }$ so we can discard it. Let us now consider the fourth term: since $d_{\omega } \tau$ is in the image of $W_1$, we can invert it and get

$$\begin{aligned} \lambda \sigma d_{\omega } \tau&= \lambda e^{N-3} \sigma W^{-1}(d_{\omega } \tau )\\&= \lambda e_n e^{N-4}d_{\omega } e W^{-1}(d_{\omega } \tau ) - \lambda e_n e^{N-4} p_{{\mathcal {T}}}(d_{\omega } e ) W_1^{-1}(d_{\omega } \tau ). \end{aligned}$$

The second term is again proportional to $R_{\tau }$ so we can discard it as well. Let us now consider the first term of this expression and $d_{\omega }(\lambda e_n)g(\tau ,\omega ,e) + \lambda \sigma g(\tau ,\omega ,e)$—the last two remaining terms. By expanding these terms using the definition of *f*, integrating by parts and using $\tau \wedge e_n = 0$ we get that these three terms add up to zero. Collecting these results, we get

$$\begin{aligned} \{ R_{\tau }, H_{\lambda } \}\approx \{R_{\tau },R_{\tau '}\} + G_{\lambda \tau } \approx F_{\tau \tau '} + G_{\lambda \tau } \not \approx 0 . \end{aligned}$$

$$\square $$

#### Remark 31

For $N=4$, some of the previous computations simplify. In particular, it is possible to give a compact explicit expression for the function $F_{\tau \tau }$. This coincides with the corresponding one of the linearized theory ${\widetilde{F}}_{\tau \tau }$ expressed in (36). As a consequence, it is also possible to give an explicit expression for the other brackets not proportional to the constraints.

#### Remark 32

As we will see in “Appendix B”, in the linearized case we can identify some first class zero modes inside the second class constraint (see Remark 46). In the nonlinearized case, such identification is more complicated but such modes should anyway be present. This will be object of future studies.

#### Corollary 33

The constraints $L_c$, $P_{\xi }$, $H_{\lambda }$ and $R_{\tau }$ do not form a first class system. In particular, $R_{\tau }$ is a second class constraint, while the others are first class (as defined in Remark 36).

#### Proof

Throughout the proof, we use the notation and terminology established in “Appendix A”. Since the bracket between $R_{\tau }$ and itself is not zero on shell, the system contains constraints that are second class. We want now to establish which constraints are of second class and which are of first class. The constraints $L_c$ and $P_{\xi }$ commute—on shell—with themselves and all the other constraints; hence, they are of first class. Let us now consider $R_{\tau }$ and $H_{\lambda }$. We want to prove that $R_{\tau }$ is of second class, while using a linear transformation of the constraints $H_{\lambda }$ is of first class. Using the result of Proposition 35, if we call *D* the matrix representing the bracket $\{R_{\tau },R_{\tau }\}$, *B* the one representing the bracket $\{ R_{\tau }, H_{\lambda } \}$, and *C* the one representing the bracket $\{H_{\lambda }, H_{\lambda }\}$, we have to prove that $B^T D^{-1} B=-C$.

From the proof of Theorem 29, we can deduce the expressions of the matrices *B*, *D* and *C*. All the components of such matrices contain a derivative in the *lightlike* direction, apart from the terms coming from $G_{\lambda \tau }$ in *B*. Hence, all components of $D^{-1}$ will contain the inverse of such derivative. Since $\lambda$ is an odd quantity, all the terms contained in $B^T D^{-1} B$ without a derivative vanish because of Lemma 50. Hence, the only surviving elements in $B^T D^{-1} B$ come from the multiplication of the elements containing a derivative in *B*. We denote such terms by $B'$. It is then a straightforward computation to check that the coefficients of such combination are actually equal to those of *C*. Indeed, since these matrices have the same functional form ($F_{\tau \tau }$), we can express the matrices $B'$ and *C*, respectively, as $B'= D p_{{\mathcal {S}}}(e_n e^{N-4} (\omega -\omega _0))$ and $C=p_{{\mathcal {S}}}(e_n e^{N-4} (\omega -\omega _0))^T D p_{{\mathcal {S}}}(e_n e^{N-4} (\omega -\omega _0))$. Hence, we have

$$\begin{aligned} B'^T D^{-1} B'&= p_{{\mathcal {S}}}(e_n e^{N-4} (\omega -\omega _0))^T D^T D^{-1} D p_{{\mathcal {S}}}(e_n e^{N-4} (\omega -\omega _0)) \\&= -p_{{\mathcal {S}}}(e_n e^{N-4} (\omega -\omega _0))^T D p_{{\mathcal {S}}}(e_n e^{N-4} (\omega -\omega _0))=-C. \end{aligned}$$

$$\square $$

We can now count the degrees of freedom of the reduced phase space. From the definition given in Section A, we can deduce that the correct number of physical degrees of freedom is given by [19, (1.60)]: let *r* be the number of degrees of freedom of the reduced phase space, *p* the number of degrees of freedom of the geometric phase space, *f* the number of first class constraints and *s* the number of second class constraints, then

$$\begin{aligned} r = p - 2f - s. \end{aligned}$$

In our case, these quantities have the following values: the geometric phase space has $2 N (N-1)$ degrees of freedom. From Corollary 33, we have that there are $\frac{N(N-1)}{2}+ N= \frac{N(N+1)}{2}$ first class constraints and $\frac{N(N-3)}{2}$ second class constraints (see Proposition 8 for the number of degrees of freedom of $\tau$). We can deduce that the correct number of local degrees of freedom is given by

$$\begin{aligned} 2 N (N-1) - N(N+1) - \frac{N(N-3)}{2} = \frac{N(N-3)}{2}. \end{aligned}$$

In the case $N=4$, this computation produces two local degrees of freedom. This result agrees with the previous works in the literature (e.g. [1]).

## Appendix A: First and Second Class Constraints

An important distinction between the constraints of a system is the one provided by the difference between first and second class constraints. In this section, we review the definition and prove a result to easily distinguish the two classes.

Roughly speaking, a constraint is of second class if its Poisson brackets with other constraints do not vanish on the constrained surfaces. However, this definition is not precise since it is always possible to take linear combinations of the constraints without modifying the reduced phase space of the theory. Furthermore, first and second class constraints correspond to different physical interpretations: the first ones are in one-to-one correspondence with the generators of gauge transformations of the theory, while the second ones are just identities through which we can express some canonical variables in terms of the other. Hence, to correctly encompass these differences, we need a more sophisticated definition. Starting from the results presented in [19, Chapter 1], we can give the following definition:

### Definition 34

Let ${\mathcal {F}}$ be a symplectic manifold, and let $\phi _i \in C^{\infty }({\mathcal {F}})$ be a set of smooth functions on it. Denote with $C_{ij}=\{\phi _i, \phi _j\}$ the matrix of the Poisson brackets of the functions. Then, the number of second class functions of the set is the rank^23^ of the matrix $C_{ij}$ on the zero locus of the functions. In particular, if $C_{ij} \approx 0$, then we say that all the functions are first class.

This definition clearly coincides with the standard one in case all the constraints are first class, i.e. all the constraints commute with every other one. However, it allows us to treat the general case, since it is invariant under rearranging the constraints by linear combinations. We now state a result that will be helpful in assessing the number of second class constraints in a system.

### Proposition 35

Let ${\mathcal {F}}$ be a symplectic manifold, and let $\psi _i,\phi _j \in C^{\infty }({\mathcal {F}})$, $i=1\dots n$, $j=1\dots m$. Denote with $C_{jj'}, B_{ij}, D_{i i'}$, respectively, the matrices representing the Poisson brackets $\{\phi _j,\phi _{j'}\}$, $\{\psi _i,\phi _j\}$ and $\{\psi _i,\psi _{i'}\}$, with $i,i'=1\dots n$, $j,j'=1\dots m$. Then, if *D* is invertible and $C= -B^T D^{-1}B$, the number of second class constraints is *n*, i.e. the rank of the matrix *D*.

### Remark 36

In this case, we will say that the $\phi$’s are the first class constraints and the $\psi$’s the second class constraints of the system.

### Proof

The matrix representing the Poisson brackets has the form

$$\begin{aligned} P= \left( \begin{array}{cc} C &{}\quad -B^T \\ B &{}\quad D \end{array} \right) \end{aligned}$$

where the blocks are as in the statement. We want to prove that this matrix is congruent to one of rank *n*, i.e. that there exists an invertible matrix *Q* such that $Q^{T}PQ$ has rank *n*. Since *D* is invertible, we can build *Q* as follows:

$$\begin{aligned} Q= \left( \begin{array}{cc} 1 &{} \quad 0 \\ -D^{-1} B &{}\quad 1 \end{array} \right) . \end{aligned}$$

An easy computation shows that

$$\begin{aligned} Q^T P Q= \left( \begin{array}{cc} C+ B^T D^{-1} B &{} \quad 0 \\ 0 &{} \quad D \end{array} \right) . \end{aligned}$$

Hence, using the second hypothesis $C=- B^T D^{-1}B$ we get the claim. $\square$

### Remark 37

This result shows explicitly that a naive definition of first class constraint as the one commuting with everything else is not sufficient to correctly consider more involved cases where the constraints do not commute (under the Poisson brackets) on the nose, but there are linear combinations of them that do. In this specific setting, from the proof of the Proposition, we gather that we can consider the set of functions

$$\begin{aligned} {\widetilde{\phi }}_j = \phi _j + \sum _{i,i'} B_{ij}D^{i i'} \psi _{i'}; \qquad {\widetilde{\psi }}_i = \psi _i \end{aligned}$$

and conclude that the functions ${\widetilde{\phi }}_j$ are first class (in the classical sense) and ${\widetilde{\psi }}_i$ are second class.

## Appendix B: Linearized Theory

In this section, we analyse the boundary structure of the linearized theory. In particular, we first introduce it on the bulk and then construct the boundary theory, respectively, in the non-degenerate and degenerate case. We present the results only in the case $N=4$. We denote with a tilde the linearized quantities to distinguish them from the general ones and use the same notation introduced in Sect. 2. The unique difference is that we will denote by $W_{e_0}^{\bullet }$ the maps $e_0 \wedge \cdot$ to highlight the difference with the normal case. The results of this appendix overlap with [26].

### B.1: Linearized Field Equations and Boundary Structure

Consider the action (3) of the Palatini–Cartan theory with the following choices of coframe and connection:

$$\begin{aligned} e&=e_0+b,\\ \omega&=\omega _0+a \end{aligned}$$

with $(e_0,\omega _0)$ a fixed solution of Euler–Lagrange equations (4) and (5) of the standard Palatini–Cartan theory. We retain only the quadratic terms in *a*, *b*; thus:

$$\begin{aligned} S_{LPC}=\int _{M}{\left( \frac{1}{2}b b F_{\omega _0}+e_0 b d_{\omega _0}a+\frac{1}{4}e_0 e_0 [a,a]{+ \frac{1}{4} \Lambda e_0e_0 bb}\right) }. \end{aligned}$$

This produces the following Euler–Lagrange equations:

25a $$\begin{aligned} e_0(d_{\omega _0}b+[a,e_0])&=0 \end{aligned}$$

25b $$\begin{aligned} b{F_{\omega _0}}+e_0 d_{\omega _0}a{+ \frac{1}{2} \Lambda e_0e_0 b}&=0. \end{aligned}$$

The first equation, as in the nonlinearized case, is equivalent to $d_{\omega _0}b+[a,e_0]=0$.

With the same procedure derived from [20] used for the general theory, we can construct the *geometric phase space* also for the linearized theory. The steps are exactly the same, while in this last case the kernel is parametrized by vector fields $X= v \frac{\delta }{\delta \omega }$ with *v* satisfying

26 $$\begin{aligned} e_0 v=0 \end{aligned}$$

instead of (10). Consequently, the *geometric* space of boundary fields of the linearized theory, $\widetilde{{\mathcal {F}}}_{LPC}$, is then parametrized by the field *b* and by the equivalence classes of *a* under the relation $a \sim a + v$ with *v* satisfying (26). The symplectic form on $\widetilde{{\mathcal {F}}}_{LPC}$ is given by

27 $$\begin{aligned} {\widetilde{\varpi }}_{LPC} = \int _{\Sigma } e_0 \delta b \delta [a]. \end{aligned}$$

The following proposition provides a shortcut (possible only in the linearized case) to the choice of a representative of the equivalence class:

#### Proposition 38

There exists a symplectomorphism $\widetilde{{\mathcal {F}}}_{LPC} \rightarrow T^*\Omega ^{1,1}_{\partial }$ equipped with the canonical symplectic form.

#### Proof

Let *b* and $\Theta$ be the fields, respectively, in the base and fibre of ${\mathcal {F}}^\partial _{LPC}=T^*\Omega ^{1,1}_{\partial }$. The symplectic form of this space is

$$\begin{aligned} \varpi ^\partial _{LPC}=\int _{\Sigma }{\delta b \delta \Theta }. \end{aligned}$$

From Lemma 4, we know that the map $W_{e_0}^{\partial ,(1,2)}$ is surjective but not injective. Hence, $\Theta$ can be written as

28 $$\begin{aligned} \Theta = e_0 a \end{aligned}$$

for some $a \in \Omega ^{1,2}_{\partial }$. Because of the definition of [*a*], it is then clear that there is a bijection between [*a*] and $\Theta$. This bijection is also a symplectomorphism since it sends the symplectic form (27) to the corresponding one of ${\mathcal {F}}^\partial _{LPC}$. $\square$

#### Remark 39

This symplectomorphism exists also in the nonlinearized case, but in both the degenerate and non-degenerate cases, it is not possible to write the action in a simple way with the new variables. Hence, this is an important feature of the linearized case.

### B.2: Non-degenerate Boundary Metric

In this section, we will implement the results of the non-degenerate theory to the linearized case. Therefore, we will consider a background boundary coframe giving rise to a non-degenerate boundary metric $g^\partial _0$. Moreover, we will compute the algebra of constraints, concluding that the reduced phase space of the linearized theory is coisotropic.

In this setting, the constraints of the theory are given by

$$\begin{aligned} e_0({d_{\omega _0}}b+[a,e_0])=0 \quad \text {and}\quad b{F_{\omega _0}}+e_0{d_{\omega _0}}a{+ \frac{1}{2} \Lambda e_0e_0 b}=0. \end{aligned}$$

Hence, using the identification (28) we can write the constraints of the boundary linearized theory as in the following definition. Let now $c \in \Omega ^{0,2}_\partial [1]$ and $\mu \in \Omega ^{0,1}_\partial [1]$ be (odd) Lagrange multipliers where the notation [1] denotes that the fields are shifted by 1 and are treated as odd variables.

The functionals defining the constraints of the non-degenerate linearized Palatini–Cartan theory are

29a $$\begin{aligned} \widetilde{L_c}&=\int _{\Sigma }{c e_0 {d_{\omega _0}}b+\Theta [c,e_0]}, \end{aligned}$$

29b $$\begin{aligned} \widetilde{J_{\mu }}&=\int _{\Sigma }{\mu \left( b{F_{\omega _0}}+{d_{\omega _0}}\Theta {+ \frac{1}{2} \Lambda e_0e_0 b}\right) } \end{aligned}$$

and the symplectic form reads

30 $$\begin{aligned} {{\widetilde{\varpi }}}^\partial =\int _{\Sigma }{\delta b \delta \Theta }. \end{aligned}$$

The constraints (29), together with the identification $\Theta = e_0 a$, are not sufficient to guarantee that ${d_{\omega _0}}b+[a,e_0]=0$. In order to get this implication, we can exploit the freedom of the choice of *a*, given by the kernel of the map $W_{e_0}$. Indeed, as it was shown in [5] for the nonlinearized case (a brief recap can be found in Sect. 3.1), for every $\Theta$ and *b* it is possible to find an *a* such that

31 $$\begin{aligned} e_n {d_{\omega _0}}b + e_n [a, e_0] \in {{\,\mathrm{Im}\,}}W_{e_0} \quad \text {and} \quad \Theta = e_0 a \end{aligned}$$

for a vector $e_n$ completing the set $e_0$ to a basis of ${\mathcal {V}}_{\Sigma }$. Then, the choice (31) together with the constraints (29) is equivalent to the restriction of the Euler–Lagrange equations on the bulk to the boundary.

We now compute the structure of the Poisson brackets of the constraints. We first need a technical lemma about the Hamiltonian vector fields of the constraints.

#### Lemma 40

The components of the Hamiltonian vector fields associated with the constraints of the non-degenerate linearized Palatini–Cartan theory are

32a $$\begin{aligned} {\mathbb {L}}_b&= [c,e_0]&{\mathbb {L}}_\Theta&= e_0{d_{\omega _0}}c \end{aligned}$$

32b $$\begin{aligned} {\mathbb {J}}_b&= {d_{\omega _0}}\mu&{\mathbb {J}}_\Theta&= \mu {F_{\omega _0}}{+ \frac{1}{2} \mu \Lambda e_0e_0 } \end{aligned}$$

where the components of a generic vector field are defined as ${\mathbb {F}}={\mathbb {F}}_b \frac{\delta }{\delta b} +{\mathbb {F}}_\Theta \frac{\delta }{\delta \Theta }$.

#### Proof

The Hamiltonian vector field ${\mathbb {F}}$ of a function *F* satisfies

$$\begin{aligned} \iota _{{\mathbb {F}}}\widetilde{\varpi }^{\partial }-\delta {{\widetilde{F}}}=0. \end{aligned}$$

The result thus follows easily from the variation of the constraints:

$$\begin{aligned} \delta \widetilde{L_c}&=\int _{\Sigma }{({d_{\omega _0}}c e_0 \delta b+[c,e_0]\delta \Theta )};\\ \delta \widetilde{J_{\mu }}&=\int _{\Sigma }{\mu \left( {F_{\omega _0}}\delta b -{d_{\omega _0}}\delta \Theta {+ \frac{1}{2} \Lambda e_0e_0 \delta b}\right) }\\ {}&=\int _{\Sigma }{\left( \mu {F_{\omega _0}}\delta b +{d_{\omega _0}}\mu \delta \Theta {+ \frac{1}{2}\mu \Lambda e_0e_0 \delta b}\right) }. \end{aligned}$$

$$\square $$

#### Theorem 41

Let $g^\partial _0$ be non-degenerate. Then, the Poisson algebra of constraints (29) is abelian, and therefore, the vanishing locus of such constraints defines a coisotropic submanifold. In particular,

33 $$\begin{aligned} \{\widetilde{L_c},\widetilde{L_c}\}=0\quad \{\widetilde{J_{\mu }},\widetilde{J_{\mu }}\}=0\quad \{\widetilde{L_c},\widetilde{J_{\mu }}\}=0. \end{aligned}$$

#### Proof

Using the definition of the Poisson bracket of a symplectic manifold

$$\begin{aligned} \{{{\widetilde{F}}},{{\widetilde{G}}}\} = \iota _{\mathbb F}\iota _{{\mathbb {G}}}\,{{\widetilde{\varpi }}}^{\partial }=\iota _{\mathbb F}\delta {{\widetilde{G}}}, \end{aligned}$$

using the results of Lemma 40, we get the following expression for the Poisson brackets of the constraints:

$$\begin{aligned} \{\widetilde{L_c},\widetilde{L_c}\}= 2 \int _{\Sigma }{[c,e_0] e_0 {d_{\omega _0}}c}=0 \end{aligned}$$

since it is the integral of a total derivative given that

$$\begin{aligned} {d_{\omega _0}}([c,c] e_0 e_0)&={d_{\omega _0}}[c,c] e_0 e_0 =2[{d_{\omega _0}}c,c] e_0 e_0\\&=2{d_{\omega _0}}c [c,e_0 e_0]=4[c,e_0] e_0 {d_{\omega _0}}c; \end{aligned}$$

$$\begin{aligned} \{\widetilde{J_{\mu }},\widetilde{J_{\mu }}\}=\int _{\Sigma }{2{d_{\omega _0}}\mu \mu {F_{\omega _0}}{+ {d_{\omega _0}}\mu \Lambda \mu e_0e_0 }}=0, \end{aligned}$$

which is equivalent to a total derivative as before, indeed

$$\begin{aligned} {d_{\omega _0}}(\mu \mu {F_{\omega _0}})&={d_{\omega _0}}(\mu \mu ) {F_{\omega _0}}+\mu \mu {d_{\omega _0}}{F_{\omega _0}}\\&={d_{\omega _0}}(\mu \mu ) {F_{\omega _0}}=2{d_{\omega _0}}\mu \mu {F_{\omega _0}}\end{aligned}$$

and $${d_{\omega _0}}e_0=0$$;

$$\begin{aligned} \{\widetilde{J_{\mu }},\widetilde{L_c}\}=\int _{\Sigma }{\left( {d_{\omega _0}}\mu e_0 {d_{\omega _0}}c+\mu {F_{\omega _0}}[c,e_0]{+ \frac{1}{2} \Lambda e_0e_0 [c, e_0]}\right) }=0, \end{aligned}$$

since

$$\begin{aligned} {d_{\omega _0}}(\mu e_0 {d_{\omega _0}}c )&= {d_{\omega _0}}\mu e_0 {d_{\omega _0}}c+\mu e_0 [{F_{\omega _0}},c] \end{aligned}$$

and $$[c, e_0^3]=0$$. $$\square $$

This proves that the reduced phase space of the non-degenerate linearized PC theory is coisotropic. This of course also follows from the linearization of the result of Reference [13] on the non-degenerate Palatini–Cartan theory.

### B.3: Degenerate Boundary Metric

Let now $g^\partial _0$ be degenerate. In this case, some of the properties useful to characterize the boundary structure of the non-degenerate case are different. In particular, from Lemma 5 the map $\varrho _0|_{\mathrm {Ker}W_{e_0}^{(1,2)}}$ is no longer injective and

$$\begin{aligned} {{\,\mathrm{Im}\,}}\left( \varrho _0|_{\mathrm {Ker}{(W_{e_0}^{(1,2)})}}\right) \ne \mathrm {Ker}{(W_{e_0}^{(2,1)})} . \end{aligned}$$

This implies that it is not possible to find an *a* that solves (31) for all $\Theta$. Digging more in the results of Sect. 2, we get that $\dim (\mathrm {Ker}{(\varrho |_{\mathrm {Ker}{(W_{e_0}^{(1,2)})}})})=2$ (Lemma 5), and consequently, $\dim ({{\,\mathrm{Im}\,}}{\varrho |_{\mathrm {Ker}{(W_{e_0}^{(1,2)})}}})=4$. Moreover, $\dim \mathrm {Ker}{(W_{e_0}^{(2,1)})}=6$, and hence, $\dim {\mathcal {T}}=2$. We conclude that if we want to be able to find *a* such that the constraints (29) are equivalent to the restriction of the Euler–Lagrange equations on the bulk to the boundary, we have to impose two extra conditions on $\Theta$ and to modify the structural constraint (31) accordingly.

Therefore, using Lemma 10, we can add to the set of constraints the additional one

34 $$\begin{aligned} {{\widetilde{R}}}_\tau = \int _{\Sigma }{\tau (d_{\omega _0}b+[a,e_0])}, \end{aligned}$$

with $\tau \in {\mathcal {S}}_0[1]$. Because of the definition^24^ of ${\mathcal {S}}_0$ and of Lemma 10, we automatically have ${{\widetilde{R}}}_\tau [b,a+v]={\widetilde{R}}_\tau [b,a]$ for $v\in \mathrm {Ker}{(W_{e_0}^{(1,2)})}$. Hence, the constraint ${{\widetilde{R}}}_\tau$ can be written in terms of $\Theta$ and *b*. Since $\int _{\Sigma }\tau [a,e_0] =- \int _{\Sigma }[\tau ,e_0] a$, we can use Lemma 11 and write ${{\widetilde{R}}}_\tau$ as

$$\begin{aligned} {{\widetilde{R}}}_\tau = \int _{\Sigma } \tau d_{\omega _0} b - W_{e_0}^{-1}([\tau ,e_0])e_0 a = \int _{\Sigma } \tau d_{\omega _0} b - W_{e_0}^{-1}([\tau ,e_0])\Theta . \end{aligned}$$

On the other hand, the structural constraint (31) is modified as follows:

35 $$\begin{aligned} e_n {d_{\omega _0}}e + e_n [a, e_0]- p_{{\mathcal {T}}}(e_n {d_{\omega _0}}e + e_n [a, e_0]) \in {{\,\mathrm{Im}\,}}W_{e_0}. \end{aligned}$$

Collecting these results, we get that the functionals defining the constraints of the linearized Palatini–Cartan theory are

$$\begin{aligned} \widetilde{L_c}&=\int _{\Sigma }c e_0 {d_{\omega _0}}b+\Theta [c,e_0];\\ \widetilde{J_{\mu }}&=\int _{\Sigma }\mu \left( b{F_{\omega _0}}+{d_{\omega _0}}\Theta {+ \frac{1}{2} \Lambda e_0e_0 b}\right) ;\\ {{\widetilde{R}}}_\tau&= \int _{\Sigma }\tau d_{\omega _0}b-W_{e_0}^{-1}([\tau ,e_0])\Theta \end{aligned}$$

where $$\tau \in {\mathcal {S}}_0[1]$$.

We can now compute the Poisson brackets of the constraints to assess their type. First, we need to compute the Hamiltonian vector field of the additional constraint $\widetilde{R_{\tau }}$:

#### Lemma 42

The components of the Hamiltonian vector field of $\widetilde{R}_\tau$ are given by

$$\begin{aligned} {\mathbb {R}}_b&= W_{e_0}^{-1}([\tau ,e_0]),&{\mathbb {R}}_\Theta&= {d_{\omega _0}}\tau . \end{aligned}$$

#### Proof

Trivial application of the equation $\iota _{{\mathbb {F}}}\widetilde{\varpi }^{\partial }-\delta {{\widetilde{F}}}=0.$ $\square$

Before proceeding to the main theorem, giving an explicit expression of the Poisson brackets of the constraints, we give some insight on Proposition 8 and of Corollary 12 in the case $N=4$.

#### Corollary 43

[of Proposition 8]. Let $p\in \Sigma$ and *U* be a neighbourhood of *p*, then in normal geodesic coordinates centred in *p* and in the standard basis of ${\mathcal {V}}_{\Sigma }$, the free components of an element $\tau \in {\mathcal {S}}_0$ are 2 and are characterized by the following equations:

$$\begin{aligned} \tau _3^{abc}&=0 \quad \forall a,b,c\\ \tau _\alpha ^{123}&=0 \quad \alpha =1,2\\ \tau _\alpha ^{124}&=0 \quad \alpha =1,2\\ \tau _2^{134}&=\frac{\tau _1^{234} {g_0}_{22}- \tau _1^{134}({g_0}_{12}+{g_0}_{21})}{{g_0}_{11}} \\ \tau _1^{134}&=-\tau _2^{234}. \end{aligned}$$

Correspondingly, we have

$$\begin{aligned} W_{e_0}^{-1}([\tau ,e_0])_1^1&= \tau _1^{134}&W_{e_0}^{-1}([\tau ,e_0])_2^2&= \tau _2^{234} \\ W_{e_0}^{-1}([\tau ,e_0])_1^2&= \tau _1^{234}&W_{e_0}^{-1}([\tau ,e_0])_2^1&= \tau _2^{134}. \end{aligned}$$

#### Theorem 44

Let $g_0^\partial$ be degenerate on $\Sigma$. Then, the structure of the Poisson brackets of the constraints $\widetilde{L_c}$, $\widetilde{J_{\mu }}$ and $\widetilde{R_{\tau }}$ is given by the following expressions:

$$\begin{aligned}&\{\widetilde{L_c}, \widetilde{L_c}\} = 0&\{\widetilde{L_c}, \widetilde{J_{\mu }}\} = 0 \\&\{\widetilde{J_{\mu }}, \widetilde{J_{\mu }}\} = 0&\{\widetilde{J_{\mu }},\widetilde{R_{\tau }}\} = {\widetilde{F}}_{\mu \tau } \\&\{\widetilde{L_c}, \widetilde{R_{\tau }}\} = 0&\{\widetilde{R_{\tau }},\widetilde{R_{\tau }}\} = {\widetilde{F}}_{\tau \tau } \end{aligned}$$

where ${\widetilde{F}}_{\mu \tau }$ and ${\widetilde{F}}_{\tau \tau }$ are functions of the background fields $e_0$, $\omega _0$ and of $\mu$ and $\tau$. These functions vanish if $\tau$ is covariantly constant.

#### Proof

The brackets between the constraints $\widetilde{L_c}$ and $\widetilde{J_{\mu }}$ are the same as in the non-degenerate case and have already been computed in Theorem 41. Let us now compute $\{\widetilde{L_c}, \widetilde{R_{\tau }}\}$. Using the results of Lemmas 40 and 42, we get

$$\begin{aligned} \{\widetilde{L_c}, \widetilde{R_{\tau }}\}&= \int _{\Sigma } [c,e_0] {d_{\omega _0}}\tau + {d_{\omega _0}}c [\tau ,e_0] = \int _{\Sigma }{d_{\omega _0}}([c,e_0] \tau ) = 0 \end{aligned}$$

where we used that ${d_{\omega _0}}e_0 =0$ and that $\Sigma$ is closed. Consider now $\{\widetilde{J_{\mu }},\widetilde{R_{\tau }}\}$:

$$\begin{aligned} \{\widetilde{J_{\mu }},\widetilde{R_{\tau }}\}&= \int _{\Sigma } {d_{\omega _0}}\mu {d_{\omega _0}}\tau + \mu {F_{\omega _0}}W_{e_0}^{-1}([\tau ,e_0]){+ \frac{1}{2} \mu \Lambda e_0[\tau ,e_0]}. \end{aligned}$$

We first note that the last term vanishes. Then, since the remaining terms do not depend on *b*, the bracket cannot be proportional to any of the constraints. We now want to prove, using coordinates, that this bracket does not vanish. Integrating by parts the first term and discarding the total derivative ($\Sigma$ closed), we get

$$\begin{aligned} \int _{\Sigma } {d_{\omega _0}}\mu {d_{\omega _0}}\tau = \int _{\Sigma } - \mu [ {F_{\omega _0}}, \tau ] = \int _{\Sigma } \tau [ {F_{\omega _0}}, \mu ]. \end{aligned}$$

We now split the computation in two parallel ways, one for the components of $\mu$ proportional to the image of $e_0$ (on the boundary) and the other for the orthogonal part of $\mu$. We parametrize $\mu$ with a vector field $\xi \in \Gamma (T\Sigma )$, such that $\mu =\iota _{\xi }e_0 + \mu ^4 e_n$. Let us denote by *F* a function of the last component $\mu ^4 e_n$. We have:

$$\begin{aligned} \{\widetilde{J_{\mu }},\widetilde{R_{\tau }}\}&= \int _{\Sigma } \tau [ {F_{\omega _0}}, \iota _{\xi } e_0 ] + \iota _{\xi } e_0 {F_{\omega _0}}W_{e_0}^{-1}([\tau ,e_0])+ F(\mu ^4 e_n)\\&= \int _{\Sigma } \tau [{F_{\omega _0}}, \iota _{\xi } e_0]+ \iota _{\xi } {F_{\omega _0}}[\tau ,e_0]+ F(\mu ^4 e_n)\\&= \int _{\Sigma } \tau \iota _{\xi }[{F_{\omega _0}}, e_0]+ F(\mu ^4 e_n) = F(\mu ^4 e_n) \end{aligned}$$

where we used that the couple $(e_0, \omega _0)$ solves the equations $e_0 {F_{\omega _0}}=0$ and ${d_{\omega _0}}e_0=0$. Consequently, the last term vanishes since $[{F_{\omega _0}},e_0]= {d_{\omega _0}}({d_{\omega _0}}e_0)= {d_{\omega _0}}(0)=0$.

In order to compute $F(\mu ^4 e_n)$, we will make use of Corollary 43 and since there are no derivatives involved here, we can also simplify the result by taking $g^{\partial }_0$ diagonal and working in the point *p* (basis point of the normal geodesic coordinates). Furthermore, this approach is also suitable for proving the same result of the tangent part, but it is way more complicated, involving the computation of all the components of the quantities appearing in the bracket. Hence, in the standard basis we have

$$\begin{aligned} \tau [ {F_{\omega _0}}, \mu ]&= \left( \tau _2^{134} [ {F_{\omega _0}}, \mu ]_{13}^{2} + \tau _1^{234} [ {F_{\omega _0}}, \mu ]_{23}^{1} \right. \\&\quad \left. - \tau _1^{134} [ {F_{\omega _0}}, \mu ]_{23}^{2} - \tau _2^{234} [ {F_{\omega _0}}, \mu ]_{13}^{1}\right) {\mathbf {V}}\\&= \left( \tau _1^{234} ([ {F_{\omega _0}}, \mu ]_{23}^{1}+ [ {F_{\omega _0}}, \mu ]_{13}^{2} )\right. \\ {}&\quad \left. - \tau _1^{134} ([ {F_{\omega _0}}, \mu ]_{23}^{2} - [ {F_{\omega _0}}, \mu ]_{13}^{1})\right) {\mathbf {V}}, \\ \mu {F_{\omega _0}}W_{e_0}^{-1}([\tau ,e_0])&= \left( -(\mu {F_{\omega _0}})_{23}^{234} \tau _{1}^{134}-(\mu {F_{\omega _0}})_{13}^{134} \tau _{2}^{234}\right) {\mathbf {V}}\\&\quad + \left( (\mu {F_{\omega _0}})_{13}^{234} \tau _{2}^{134}+ (\mu {F_{\omega _0}})_{23}^{134} \tau _{1}^{234}\right) {\mathbf {V}} \\&= \left( \tau _{1}^{134}( (\mu {F_{\omega _0}})_{23}^{234} -(\mu {F_{\omega _0}})_{13}^{134})\right) {\mathbf {V}} \\&\quad - \left( \tau _{1}^{234}((\mu {F_{\omega _0}})_{23}^{134} +(\mu {F_{\omega _0}})_{13}^{234})\right) {\mathbf {V}} \end{aligned}$$

where ${\mathbf {V}}= {e_0}_1 {e_0}_2 {e_0}_3 e_n dx^1 dx^2 dx^3$. Using the two identities

$$\begin{aligned}{}[ {F_{\omega _0}}, \mu ]_{ab}^{i}= \sum _{k,j}{F_{\omega _0}}_{ab}^{ij}\eta _{jk} \mu ^k \; \text {and} \; (\mu {F_{\omega _0}})_{ab}^{ijk}= \mu ^i {F_{\omega _0}}_{ab}^{jk} + \mu ^j {F_{\omega _0}}_{ab}^{ki} + \mu ^k {F_{\omega _0}}_{ab}^{ij} \end{aligned}$$

, we can further simplify $\tau [ {F_{\omega _0}}, \mu ] + \mu {F_{\omega _0}}W_{e_0}^{-1}([\tau ,e_0])$. A simple computation shows (as it should be because of the first part of this proof) that the terms containing $\mu ^3$ are the same with opposite sign in the two summands; hence, they cancel and the terms containing $\mu ^1$ and $\mu ^2$ vanish because they contain components of ${F_{\omega _0}}$ that are zero due to the equations $e_0 {F_{\omega _0}}=0$ and ${d_{\omega _0}}e_0=0$. Hence, we are left with the terms containing $\mu ^4 e_n$ and get

$$\begin{aligned} F(\mu ^4 e_n)&= \left( \tau _1^{234} ({F_{\omega _0}}_{23}^{13} \mu ^4 + {F_{\omega _0}}_{13}^{23} \mu ^4) - \tau _1^{134}({F_{\omega _0}}_{23}^{23}\mu ^4 -{F_{\omega _0}}_{13}^{13}\mu ^4)\right) {\mathbf {V}}\\&\quad + \left( \tau _1^{234} (\mu ^4 {F_{\omega _0}}_{23}^{13}+ \mu ^4 {F_{\omega _0}}_{13}^{23}) - \tau _1^{134}(\mu ^4 {F_{\omega _0}}_{23}^{23}-\mu ^4 {F_{\omega _0}}_{13}^{13})\right) {\mathbf {V}}\\&= 2 \mu ^4\left( -\tau _1^{234} ({F_{\omega _0}}_{23}^{13} + {F_{\omega _0}}_{13}^{23} ) + \tau _1^{134}({F_{\omega _0}}_{23}^{23} -{F_{\omega _0}}_{13}^{13})\right) {\mathbf {V}}. \end{aligned}$$

These expression does not vanish since none of the equations $e_0 {F_{\omega _0}}=0$ and ${d_{\omega _0}}e_0=0$ contain these components of ${F_{\omega _0}}$. We denote by ${\widetilde{F}}_{\mu \tau }= F(\mu ^4 e_n)$ and note that if ${d_{\omega _0}}\tau =0$ (i.e. $\tau$ covariantly constant) it vanishes. Let us now pass to the last bracket:

$$\begin{aligned} \{\widetilde{R_{\tau }},\widetilde{R_{\tau }}\}&= \int _{\Sigma } W_{e_0}^{-1}([\tau ,e_0]){d_{\omega _0}}\tau . \end{aligned}$$

As in the previous case, this bracket does not depend on *b* and $\Theta$, hence it cannot be proportional to any constraint. Again, we want to prove that this expression does not vanish and in order to reach this goal, we will work in coordinates using the results of Corollary 43. Note that since the expression of the bracket entails taking derivatives, we need to work in a neighbourhood and retain the complete expression for the relations between the components of $\tau$.

36 $$\begin{aligned} \{\widetilde{R_{\tau }},\widetilde{R_{\tau }}\}&=\int _{\Sigma } \left( -\tau _1^{134} \partial _3 \tau _2^{234}-\tau _2^{234} \partial _3 \tau _1^{134}+ \tau _2^{134} \partial _3 \tau _1^{234}+ \tau _1^{234} \partial _3 \tau _2^{134}\right) {\mathbf {V}} \nonumber \\&= \int _{\Sigma }\left( 2\tau _1^{134} \partial _3 \tau _1^{134}+ \tau _1^{234} \partial _3 \tau _1^{234}+ \tau _1^{234} \partial _3 \frac{\tau _1^{234} {g_0}_{22}-2 \tau _1^{134}{g_0}_{12}}{{g_0}_{11}}\right) {\mathbf {V}}. \end{aligned}$$

For a generic (not covariantly constant) $\tau$ and a generic background metric $g_0$, this expression does not vanish. We denote this last quantity with ${\widetilde{F}}_{\tau \tau }$. $\square$

#### Corollary 45

If $\tau$ is not covariantly constant, the constraints $\widetilde{L_c}$, $\widetilde{J_{\mu }}$ and $\widetilde{R_{\tau }}$ do not form a first class system. In particular, $\widetilde{R_{\tau }}$ is a second class constraint, while the others are first class.

#### Proof

We prove this result using Proposition 35 and subsequently by trivially applying Lemma 50. $\square$

#### Remark 46

In case $\tau$ is covariantly constant, i.e. $d_{\omega _0} \tau =0$, also the constraint $\widetilde{R_{\tau }}$ is first class. Indeed, we get

$$\begin{aligned} \{\widetilde{R_{\tau }},\widetilde{R_{\tau }}\}&= \int _{\Sigma } W_{e_0}^{-1}([\tau ,e_0]){d_{\omega _0}}\tau =0. \end{aligned}$$

These are usually called first-class zero mode [1]. The interpretation of these zero modes in terms of symmetries is still unknown and will be the object of future studies.

These results can be compared with the ones in [17] where the same problem is analysed in the Einstein–Hilbert formalism. In this article, the authors found 2*N* first class constraints (shared with the time/spacelike case) and additional $N(N-3)/2$ second class constraints. Despite the different number of first-class constraints, the results here outlined coincide exactly with those that we have found. Indeed, we found exactly the same amount of additional second-class constraints, while the difference in the number of first-class constraint is due to the different formalism adopted. Indeed, in the space- or timelike case in the Einstein–Hilbert formalism one has 2*N* first-class constraints [11], while in the Palatini–Cartan formalism, one has $N(N+1)/2$ first-class constraints [5]. This difference is due to the different number of degrees of freedom of the space of boundary fields. Note, however, that the numbers of physical degrees of freedom are the same. The actual comparison of the results of the present article and those in [17] can be made more precise through a procedure similar to that outlined in [13, Theorem 4.25] and will be the object of future work.

## Appendix C: Long Proofs

All the proofs of Lemmas 3 and 4 can be found in [5, Appendix A]. We recall here verbatim the proof of point (3) of Lemma 4 in order to have a useful reference for the new proofs.

### Proof of Lemma 4.(3)

([5]) Consider $$W_{N-3}^{ \partial , (1,2)}:\Omega _{N, \partial }^{1,2} \longrightarrow \Omega _{N, \partial }^{N-2,N-1}$$.

The dimensions of domain and codomain of this map are, respectively, $\dim \Omega _{N, \partial }^{1,2} = (N-1)\frac{N(N-1)}{2}$ and $\dim \Omega _{N, \partial }^{N-2,N-1} = (N-1)N$. The kernel of $W_{N-3}^{ \partial , (1,2)}$ is defined by the following set of equations:

$$\begin{aligned} X_{\mu _1}^{ab} e_a e_b \wedge e_{\mu _2} \wedge \dots \wedge e_{\mu _{N-2}} dx^{\mu _1}dx^{\mu _2}\dots dx^{\mu _{N-2}}=0 \end{aligned}$$

where we used $e_a$ as a basis for ${\mathcal {V}}_{\Sigma }$. Let now $k=N$ be the transversal direction, and let $k'\in \{1, \dots N-1\}$. Since $\{dx^{\mu _1}dx^{\mu _2}\dots dx^{\mu _{N-2}}\}$ is a basis for $\Omega _{\partial }^{N-2}$, we obtain $N-1$ equations of the form

$$\begin{aligned} \sum _{\sigma } X_{\mu _{\sigma (1)}}^{ab} e_a e_b \wedge e_{\mu _{\sigma (2)}} \wedge \dots \wedge e_{\mu _{\sigma (N-2)}} =0 \end{aligned}$$

where $\sigma$ runs on all permutations of $N-2$ elements and $1\le \mu _i \le N-1$, $\mu _i \ne k'$ for all $1\le i \le N-2$. Recall now that $e_a e_b \wedge e_{\mu _{\sigma (2)}} \wedge \dots \wedge e_{\mu _{\sigma (N-2)}}$ is a basis of $\wedge ^{N-1}{\mathcal {V}}_{\Sigma }$. Hence, we obtain the following equations:

$$\begin{aligned} X_{i}^{N k'} =0 \qquad&1 \le i \le N-1 \; i \ne k', \\ \sum _{i\ne N, i\ne k'} X_{i}^{iN} =0, \qquad&\sum _{i\ne N, i\ne k'} X_{i}^{ik'} =0 . \end{aligned}$$

Letting now $k'$ vary in $\{1, \dots , N-1\}$, we obtain the following equations:

37a $$\begin{aligned} X_{i}^{N j} =0 \qquad&1 \le i,j \le N-1 \; i \ne j ,\end{aligned}$$

37b $$\begin{aligned} \sum _{i\ne N, i\ne j} X_{i}^{iN} =0 \qquad&1 \le j \le N-1, \end{aligned}$$

37c $$\begin{aligned} \sum _{i\ne N, i\ne j} X_{i}^{ij} =0 \qquad&1 \le j \le N-1 . \end{aligned}$$

It is easy to check that these equations are independent. The total number of equations defining the kernel is then $(N-1)+(N-1)(N-2)+(N-1) = (N-1)N$ which coincides with number of degrees of freedom of the codomain. Hence, $W_{N-3}^{ \partial , (1,2)}$ is surjective but not injective. In particular, $\dim \text {Ker} W_{N-3}^{\partial , (1,2)}= (N-1)\frac{N(N-1)}{2}- N(N-1)= \frac{N(N-1)}{2}(N-3)$. $\square$

### Proof of Lemma 5

Consider $\varrho |_{\text {Ker} W_{N-3}^{\partial , (1,2)}}: \text {Ker} W_{N-3}^{\partial , (1,2)} \rightarrow \Omega _{N, \partial }^{2,1}$. From the proof of Lemma 4.(3), we know that $\dim \text {Ker} W_{N-3}^{\partial , (2,1)}= \frac{N(N-1)}{2}(N-3)$. An element $v \in \text {Ker} W_{N-3}^{\partial , (1,2)}$ must satisfy the following equations:

38a $$\begin{aligned} v_{i}^{N j} =0 \qquad&1 \le i,j \le N-1 \; i \ne j, \end{aligned}$$

38b $$\begin{aligned} \sum _{i\ne N, i\ne j} v_{i}^{iN} =0 \qquad&1 \le j \le N-1, \end{aligned}$$

38c $$\begin{aligned} \sum _{i\ne N, i\ne j} v_{i}^{ij} =0 \qquad&1 \le j \le N-1. \end{aligned}$$

The kernel of $\varrho$ is defined by the following set of equations^25^:

$$\begin{aligned}{}[v,e]_{\mu _1\mu _2}^a= v_{\mu _1}^{ab} g^{\partial }_{b \mu _2} - v_{\mu _2}^{ab}g^{\partial }_{b\mu _1}=0. \end{aligned}$$

Using now normal geodesic coordinates, we can diagonalize $g^\partial$ with eigenvalues on the diagonal $\alpha _{\mu } \in \{1,-1,0\}$:

39 $$\begin{aligned}{}[v,e]_{\mu _1\mu _2}^a= v_{\mu _1}^{a\mu _2} \alpha _{ \mu _2} - v_{\mu _2}^{a\mu _1} \alpha _{\mu _1}=0. \end{aligned}$$

Let now $\alpha _{ \mu }=0$ for $\mu = N-1$ and $\alpha _{ \mu }= \pm 1$ for $1 \le \mu \le N-2$. We adopt now the following convention on indices for $m,p\in {\mathbb {N}}$: $1 \le i_m \le N-2$, $i_m \ne i_p$ iff $m\ne p$. Using $v \in \text {Ker} W_{N-3}^{\partial , (2,1)}$, from (39) we get

40a $$\begin{aligned}{}[v,e]_{i_1 i_2}^{i_3}&= v_{i_1}^{ i_3 i_2} - v_{i_2}^{ i_3 i_1}&[v,e]_{i_1 i_2}^{i_1}&= v_{i_1}^{i_1 i_2} \end{aligned}$$

40b $$\begin{aligned} _{i_1 i_2}^{N-1}&= v_{i_1}^{N-1 i_2} - v_{i_2}^{N-1 i_1}&[v,e]_{i_1 i_2}^{N}&= 0 \end{aligned}$$

40c $$\begin{aligned} _{i_1 N-1}^{i_2}&= - v_{N-1}^{i_2 i_1}&[v,e]_{i N-1}^{N-1}&= - v_{N-1}^{N-1 i} \end{aligned}$$

40d $$\begin{aligned} _{i N-1}^{i}&=0&[v,e]_{i N-1}^{N}&= 0. \end{aligned}$$

By imposing that every component vanishes, we get the corresponding equations for the kernel. It is easy to check that these equations are independent but the second of (40a) and the first of (40c) which are connected by (38c). The total number of equations defining the kernel is then

$$\begin{aligned} \frac{(N-2)(N-3)(N-4)}{2} +(N-3)(N-4)+ (N-2)(N-3). \end{aligned}$$

Since $\frac{N(N-1)}{2}(N-3)$ is the number of degrees of freedom of the domain, the kernel has dimension

$$\begin{aligned} \dim (\text {Ker}\varrho |_{\mathrm {Ker} W_{N-3}^{\partial , (1,2)}})= \frac{N(N-3)}{2}. \end{aligned}$$

$$\square $$

### Proof of Proposition 8

We split the proof into simpler lemmas. First, we compute the dimension of ${\mathcal {S}}$ and the equations defining it at the point *p* in Lemmas 47 and 48 .

Collecting these results, we get that ${\mathcal {S}}$ is defined by the following equations:

$$\begin{aligned} \sum _{\mu _1 \dots \mu _{N-3}=1}^{N-2} X_{\mu _1 \dots \mu _{N-3}}^{N N-1 \mu _1 \dots \mu _{N-3}}=0&\\ X_{N-1 i_{1}\dots i_{n-4}}^{\mu _1 \dots \mu _{N-1}}=0&\quad 1 \le \mu _a \le N, \; 1 \le i_a \le N-2 \\ X_{ i_1 \dots i_{N-3}}^{N i_1 \dots i_{N-2}}=0&\quad 1 \le i_a \le N-2 \\ X_{ i_1 \dots i_{N-3}}^{N-1 i_1 \dots i_{N-2} }=0&\quad 1 \le i_a \le N-2 \\ X_{i_1\dots i_{N-4} i_{N-3}}^{N N-1 i_1\dots i_{N-4} i_{N-2}}+X_{i_1\dots i_{N-4} i_{N-2}}^{N N-1 i_1\dots i_{N-4} i_{N-3}}=0&\quad 1 \le i_a \le N-2. \end{aligned}$$

Counting them and subtracting the result to the total dimension of $\Omega ^{N-3,N-1}_{\partial }$, we get the claimed result. Then, in Lemma 49 we express the equations defining the kernel of ${\widetilde{\varrho }}^{(N-3, N-1)}$ in the geodesic neighbourhood *U* in terms of the components of $\tau \in {\mathcal {S}}$ and those of the modified metric ${\widetilde{g}}^{\partial } {:=} \eta ({\widetilde{e}},{\widetilde{e}})$ and find the corresponding free components. Note also that the equations in Lemma 47 hold in a neighbourhood since we are not using normal geodesic coordinates in the proof. $\square$

### Lemma 47

The space $\mathrm {Ker}W_{1}^{\partial (N-3,N-1)} \subset \Omega ^{N-3,N-1}_{\partial }$ in the standard basis is defined by the following $N-1$ equations

$$\begin{aligned} \sum _{\begin{array}{c} \mu _1 \dots \mu _{N-3}=1\\ \mu _i \ne k, \mu _i \ne \mu _j \end{array}}^{N-1} X_{\mu _1 \dots \mu _{N-3}}^{N k \mu _1 \dots \mu _{N-3}} =0 \qquad 1 \le k \le N-1. \end{aligned}$$

### Proof

Consider $W_{1}^{ \partial , (N-3,N-1)}: \Omega _{\partial }^{N-3,N-1} \longrightarrow \Omega _{\partial }^{N-2,N}$: the kernel of it is defined by the following set of equations:

$$\begin{aligned} X_{\mu _1 \dots \mu _{N-3}}^{a \dots a_{N-1}} e_a \wedge \dots \wedge e_{a_{N-1}} \wedge e_{\mu _{N-2}} dx^{\mu _1}dx^{\mu _2}\dots dx^{\mu _{N-2}}=0 \end{aligned}$$

where we used $e_a$ as a basis for ${\mathcal {V}}_{\Sigma }$. Since $\{dx^{\mu _1}dx^{\mu _2}\dots dx^{\mu _{N-2}}\}$ is a basis for $\Omega _{\partial }^{N-2}$, we obtain $N-1$ equations of the form

$$\begin{aligned} \sum _{\sigma } X_{\mu _{\sigma (1)} \dots \mu _{\sigma (N-3)}}^{a \dots a_{N-1}} e_a \wedge \dots \wedge e_{a_{N-1}} \wedge e_{\mu _{\sigma (N-2)}} =0 \end{aligned}$$

where $\sigma$ runs on all permutations of $N-2$ elements and $1\le \mu _i \le N-1$ and denote by *k* the missing index: $\mu _i \ne k$ for all $1\le i \le N-2$. Recall now that $e_a \wedge e_{\mu _{\sigma (2)}} \wedge \dots \wedge e_{\mu _{\sigma (N-2)}}$ is a basis of $\wedge ^{N} {\mathcal {V}}_{\Sigma }$. Hence, we obtain the following $N-1$ equations:

$$\begin{aligned} \sum _{\begin{array}{c} \mu _1 \dots \mu _{N-3}=1\\ \mu _i \ne k, \mu _i \ne \mu _j \end{array}}^{N-1} X_{\mu _1 \dots \mu _{N-3}}^{N k \mu _1 \dots \mu _{N-3}} =0 \qquad 1 \le k \le N-1. \end{aligned}$$

$$\square $$

### Lemma 48

The space $\mathrm {Ker} {\tilde{\rho }} |_{\Omega ^{N-3,N-1}_{\partial }} \subset \Omega ^{N-3,N-1}_{\partial }$ is defined by the following four sets of equations in the standard basis:

$$\begin{aligned} X_{N-1 i_{1}\dots i_{n-4}}^{\mu _1 \dots \mu _{N-1}}=0&\quad 1 \le \mu _a \le N, \; 1 \le i_a \le N-2 \\ X_{ i_1 \dots i_{N-3}}^{N i_1 \dots i_{N-2} }=0&\quad 1 \le i_a \le N-2 \\ X_{i_1 \dots i_{N-3}}^{ i_1 \dots i_{N-2} N-1}=0&\quad 1 \le i_a \le N-2 \\ X_{ i_1\dots i_{N-4} i_{N-3}}^{N N-1 i_1\dots i_{N-4} i_{N-2}} +&X_{ i_1\dots i_{N-4} i_{N-2}}^{N N-1 i_1\dots i_{N-4} i_{N-3}} =0 \quad 1 \le i_a \le N-2. \end{aligned}$$

### Proof

In normal geodesic coordinates, the boundary metric $g^\partial$ at the base point is diagonal, and we can assume that its eigenvalues $\alpha _i$ are such that $\alpha _a= 1$ for $1\le a \le N-2$ and $\alpha _{N-1}=0$. Since *X* is such that $\iota _X g^\partial =0$, we get $X = \partial _{N-1}$. Let now be $\beta = \sum _{i=1}^{N-1} \beta _i dx^i$ a generic one form. From the equation $\iota _X \beta =1$, we get $\beta _{N-1}=1$. Hence, ${\widehat{e}}= \sum _{i=1}^{N-1} \beta _i dx^i e_{N-1}$ and ${\widetilde{e}}= \sum _{i=1}^{N-2}(e_{i} + \beta _i e_{N-1})dx^{i}$. We now impose the last condition to find an explicit expression for ${\widetilde{e}}$ in the standard basis.

Using these coordinates, since $X = \partial _{N-1}$, we can take $Y^i_0$ as $Y^i_0=\partial _i$. Let now $v \in \Omega _{\partial }^{1,2}$ such that $e^{N-3}v=0$, i.e. its components must satisfy (38). Using the same techniques as in the proof of Lemma 4.(3), we get

$$\begin{aligned} v e^{N-4} {\widetilde{e}}&= X_{\mu _1}^{ab} e_a e_b \wedge e_{\mu _2} \wedge \dots \wedge e_{\mu _{N-3}} {\widetilde{e}}_{\mu _{N-2}}^c e_c dx^{\mu _1}dx^{\mu _2}\dots dx^{\mu _{N-2}}\\&= X_{\mu _1}^{ab} e_a e_b \wedge e_{\mu _2} \wedge \dots \wedge e_{\mu _{N-3}} e_{\mu _{N-2}} dx^{\mu _1}dx^{\mu _2}\dots dx^{\mu _{N-2}}\\&\quad - X_{\mu _1}^{ab} e_a e_b \wedge e_{\mu _2} \wedge \dots \wedge e_{\mu _{N-3}} \beta _{\mu _{N-2}}e_{N-1} dx^{\mu _1}dx^{\mu _2}\dots dx^{\mu _{N-2}} \end{aligned}$$

where in the second and third line $\mu _{N-2}$ cannot take the value $N-1$. Equating this quantity to zero, we get the following equations for the components:

$$\begin{aligned} \sum _{\sigma } X_{\mu _{\sigma (1)}}^{ab} e_a e_b e_{\mu _{\sigma (2)}} \dots e_{\mu _{\sigma (N-2)}}\!-\! X_{\mu _{\sigma (1)}}^{ab} e_a e_b e_{\mu _{\sigma (2)}} \dots e_{\mu _{\sigma (N-3)}} e_{N-1} \beta _{\mu _{\sigma (N-2)}}\!=\!0 \end{aligned}$$

where $\mu _{\sigma (N-2)} \ne N-1$. Now, letting

$$\{\mu _1 \dots \mu _{N-2}\}=\{1 \dots N-2\}$$ we get

$$\begin{aligned}&\sum _{i\ne j,N-1,N} \left( X_{j}^{N-1 N} - X_{j}^{iN} \beta _i + X_{i}^{iN} \beta _j\right) =0 \qquad j = 1 \dots N-2\\&\sum _{i\ne N-1,N} X_{i}^{i N} =0 \\&\sum _{i\ne N-1,N} X_{i}^{i N-1}- \sum _{i,j\ne N-1,N} X_{i}^{i j}\beta _j =0. \end{aligned}$$

Using the properties (38), we can deduce from the very first equation that $\beta _i=0$ for $i=1 \dots N-2$. Plugging this result into the others, we do not get any further condition, as all the quantities vanish automatically. We deduce that, with this choice of the coordinates $x_i$, ${\widetilde{e}}= \sum _{i=1}^{N-2}e_{i}dx^i$.

Now, using the same procedure as in Lemma 5 we obtain the following equations defining the kernel of ${\tilde{\rho }}$:

$$\begin{aligned}{}[\tau , e]_{\mu _1 \dots \mu _{N-2}}^{\nu _1 \dots \nu _{N-2}}= \sum _{\sigma _{N-2}} \tau _{\mu _{\sigma (1)}\dots \mu _{\sigma (N-3)}}^{\nu _1 \dots \nu _{N-2}\mu _{\sigma (N-2)}} \alpha _{\mu _{\sigma (N-2)}} = 0 \end{aligned}$$

where $1\le \mu _a \le N-1$, $1\le \nu _a \le N$, $\alpha _a=1$ for $1\le a \le N-2$, $\alpha _{N-1}=0$ and $\sigma _{N-2}$ represents the permutation of $N-2$ elements. Using the properties of the $\alpha$s, we get

41a $$\begin{aligned} \sum _{\sigma _{N-3}} \tau _{N-1 i_{\sigma (1)}\dots i_{\sigma (N-4)}}^{\nu _1 \dots \nu _{N-2}i_{\sigma (N-3)}} = 0&\quad 1 \le i_a \le N-2 \end{aligned}$$

41b $$\begin{aligned} \sum _{\sigma _{N-2}} \tau _{i_{\sigma (1)}\dots i_{\sigma (N-3)}}^{\nu _1 \dots \nu _{N-2}i_{\sigma (N-2)}} = 0&\quad 1 \le i_a \le N-2 \end{aligned}$$

for $1 \!\le \! \nu _a \!\le \! N$. Let us consider the first set of equations. If $\{\nu _1, \dots ,\nu _{N-2} \} \!\supset \{i_1, \dots ,i_{N-3} \}$, no term survives and we do not get equations. Let now *n* be an index in $\{i_1, \dots ,i_{N-3} \}$ but not in $\{\nu _1, \dots ,\nu _{N-2} \}$: then, only one term survives and we have the following equations:

$$\begin{aligned} \tau _{N-1 i_{1}\dots i_{N-4}}^{\nu _1 \dots \nu _{N-2}n} = 0 \end{aligned}$$

where $1 \le i_a, n \le N-2$ and $\{\nu _1, \dots ,\nu _{N-2} \} \supset \{i_1, \dots ,i_{N-4} \}$. The only other case that is left is when there are two indices $n_1, n_2$ in $\{i_1, \dots ,i_{N-3} \}$ but not in $\{\nu _1, \dots ,\nu _{N-2} \}$: here, two terms of the sum are surviving and we get:

$$\begin{aligned} \tau _{N-1 i_{1}\dots i_{N-5}n_1}^{N N-1 i_{1}\dots i_{N-5} n_3 n_2} + \tau _{N-1 i_{1}\dots i_{N-5}n_2}^{N N-1 i_{1}\dots i_{N-5} n_3 n_1} = 0 \end{aligned}$$

where $1 \le i_a, n \le N-2$ and $n_3$ is the only index left different from all the others. Because of the arbitrariness of $n_1, n_2, n_3$, this set of equations will contain also the ones corresponding to permutations of them:

$$\begin{aligned}&\tau _{N-1 i_{1}\dots i_{N-5}n_1}^{N N-1 i_{1}\dots i_{N-5} n_2 n_3} + \tau _{N-1 i_{1}\dots i_{N-5}n_3}^{N N-1 i_{1}\dots i_{N-5} n_2 n_1} = 0 \\&\tau _{N-1 i_{1}\dots i_{N-5}n_2}^{N N-1 i_{1}\dots i_{N-5} n_1 n_3} + \tau _{N-1 i_{1}\dots i_{N-5}n_3}^{N N-1 i_{1}\dots i_{N-5} n_1 n_2} = 0 . \end{aligned}$$

Composing these three equations, we get that

$$\begin{aligned} \tau _{N-1 i_{1}\dots i_{N-5}n_1}^{N N-1 i_{1}\dots i_{N-5} n_3 n_2}=0. \end{aligned}$$

Together with the first case, this proves the first set of equations in the statement. We proceed in the same way for the second set in (41). If $\{\nu _1, \dots ,\nu _{N-2} \} \!\supset \{i_1, \dots ,i_{N-3} \}$, no term survives and we do not get equations. Let now *n* be an index in $\{i_1, \dots ,i_{N-3} \}$ but not in $\{\nu _1, \dots ,\nu _{N-2} \}$. We get

$$\begin{aligned} X_{ i_1 \dots i_{N-3}}^{N i_1 \dots i_{N-3} n}=0&\quad 1 \le i_a \le N-2 \\ X_{ i_1 \dots i_{N-3}}^{N-1 i_1 \dots i_{N-3} n}=0&\quad 1 \le i_a \le N-2 \end{aligned}$$

which are, respectively, the second and the third set of equations in the statement. When there are two indices $n_1, n_2$ in $\{i_1, \dots ,i_{N-3} \}$ but not in $\{\nu _1, \dots ,\nu _{N-2} \}$, we get the fourth set of equations:

$$\begin{aligned} X_{ i_1\dots i_{N-4} n_1}^{N N-1 i_1\dots i_{N-4} n_2} +&X_{ i_1\dots i_{N-4} n_2}^{N N-1 i_1\dots i_{N-4} n_1} =0 \quad \quad 1 \le i_a \le N-2. \end{aligned}$$

$$\square $$

### Lemma 49

Let $p \in \Sigma$ and *U* an open neighbourhood of *p*. Then, in the standard basis of ${\mathcal {V}}_{\Sigma }$, the equations defining the space $\mathrm {Ker} {\tilde{\rho }} |_{\Omega ^{N-3,N-1}_{\partial }} \subset \Omega ^{N-3,N-1}_{\partial }$ are

$$\begin{aligned} X_{N-1 i_{1}\dots i_{n-4}}^{\mu _1 \dots \mu _{N-1}}=0&\quad 1 \le \mu _a \le N, \; 1 \le i_a \le N-2 \\ X_{ i_1 \dots i_{N-3}}^{N i_1 \dots i_{N-2}}=0&\quad 1 \le i_a \le N-2 \\ X_{ i_1 \dots i_{N-3}}^{N-1 i_1 \dots i_{N-2} }=0&\quad 1 \le i_a \le N-2 \\ X_{i_1\dots i_{N-4} i_{N-3}}^{N N-1 i_1\dots i_{N-4} i_{N-2}} =&f({\widetilde{g}}^{\partial }, X_{i_1\dots i_{N-4} i_{N-2}}^{N i_1\dots i_{N-4} N-1 i_{N-3}}, X_{i_1\dots i_{N-3}}^{N N-1 i_1\dots i_{N-3}}) \end{aligned}$$

for some function *f*.

### Proof

Using the standard basis of ${\mathcal {V}}_{\Sigma }$, we obtain the following equations for the kernel of ${\tilde{\rho }}$:

42 $$\begin{aligned}{}[\tau , {\tilde{e}}]_{j_1 \dots j_{N-2}}^{i_1 \dots i_{N-2}}= \sum _{\sigma , \mu } \tau _{j_{\sigma (1)}\dots j_{\sigma (N-3)}}^{i_1 \dots i_{N-2}\mu } {\widetilde{g}}^{\partial }_{\mu j_{\sigma (N-2)}}=0 \end{aligned}$$

where $\sigma$ runs over the permutations of order $N-2$ and $\mu =1 \dots N-2$, $i_k \in \{1\dots N-1\}$, $j_k \in \{1\dots N\}$. Using normal geodesic coordinates, ${\widetilde{g}}^{\partial }$ is diagonal in the point *p*, with diagonal entries different from zero. Hence, using continuity, in the whole neighbourhood *U* (eventually shrinking it if necessary) the diagonal component will be nonzero. Furthermore, $\det {\widetilde{g}}^{\partial }\ne 0$, since ${\widetilde{g}}^{\partial }$ is non-degenerate by construction.

We first analyse the case when $N-1 \in \{i_1, \dots , i_{N-2}\}$ and prove the first set of equations in the statement. Expanding the equations (42) in all possible choices of indexes, one finds a overdetermined system of equations, and expressing it in its matricial form, it is always possible to find a square submatrix whose determinant is equal to $\det {\widetilde{g}}^{\partial }\ne 0$. This implies that all the variables must be zero.

Let now $N-1 \notin \{i_1, \dots , i_{N-2}\}$. If $N, N-1 \notin \{j_1, \dots , j_{N-2}\}$, no equations are generated. Let then $N \in \{j_1, \dots , j_{N-2}\}$ or $N-1 \in \{j_1, \dots , j_{N-2}\}$ but not $N, N-1 \in \{j_1, \dots , j_{N-2}\}$. We proceed as in the previous case and obtain a system of equations whose only solution is the zero one. Hence, we deduce the second and the third set of equations in the statement. Let now $N, N-1 \in \{j_1, \dots , j_{N-2}\}$. Expanding equations (42), we get

$$\begin{aligned}{}[\tau , {\tilde{e}}]^{N N-1 \mu _3 \dots \mu _{N-2}}_{\mu _1 \mu _2 \mu _3 \dots \mu _{N-2}}= \sum _{\sigma }&\tau _{\mu _{\sigma (1)}\dots \mu _{\sigma (N-3)}}^{N N-1 \mu _3 \dots \mu _{N-2}\mu _1} {\widetilde{g}}^{\partial }_{\mu _1 \mu _{\sigma (N-2)}} \\ +&\tau _{\mu _{\sigma (1)}\dots \mu _{\sigma (N-3)}}^{N N-1 \mu _3 \dots \mu _{N-2}\mu _2} {\widetilde{g}}^{\partial }_{\mu _2 \mu _{\sigma (N-2)}}=0. \end{aligned}$$

Inverting some of the equation exploiting the properties of ${\widetilde{g}}^{\partial }$, we can express the components $\tau _{\mu _3\dots \mu _{(N-3)}\mu _2}^{N N-1 \mu _3 \dots \mu _{N-2}\mu _1}$ with $\mu _1 < \mu _2$ in function of the components of ${\widetilde{g}}^{\partial }$, $\tau _{\mu _3\dots \mu _{(N-3)}\mu _1}^{N N-1 \mu _3 \dots \mu _{N-2}\mu _2}$ (with $\mu _1 < \mu _2$) and $\tau _{\mu _2\dots \mu _{(N-3)}}^{N N-1 \mu _2 \dots \mu _{N-3}}$.$\square$

### Proof of Lemma 10

From the proof of Lemma 4.(3), the free components of an element in ${\mathcal {T}}$ are:

$$\begin{aligned}&X^{i_1}_{N-1 i_2} \quad 1 \le i_1, i_2 \le N-2, \; i_1 \ne i_2\\&X^{i}_{i N-1} \quad 1 \le i \le N-2 \end{aligned}$$

such that

$$\begin{aligned} X^{i_1}_{j i_2} = - X^{i_2}_{j i_1}; \quad \sum _{\mu =1}^{N-1} X^{\mu }_{\mu j} =0. \end{aligned}$$

From Proposition 8, the free components of an element $\tau \in {\mathcal {S}}$ are $Y_{\mu }$ and $X_{\mu _1}^{\mu _2}$ satisfying

$$\begin{aligned} \sum _{\mu =1}^{N-2} Y_{\mu } =0 \text { and } X_{\mu _1}^{\mu _2} =- X_{\mu _2}^{\mu _1} \end{aligned}$$

for $\mu _1, \mu _2= 1 \dots N-2$. Let us now consider some particular choices of $\tau$. First, we consider $\tau$ such that the only nonzero components are $\tau _{\mu _1}^{\mu _2}=-\tau _{\mu _2}^{\mu _1}$ for some particular $\mu _1$ and $\mu _2$. Then,

$$\begin{aligned} \int _{\Sigma } \tau \alpha&= \int _{\Sigma }( X_{\mu _1}^{\mu _2} \alpha ^{\mu _1}_{N-1 \mu _2} + X_{\mu _2}^{\mu _1} \alpha ^{\mu _2}_{N-1 \mu _1}){\mathbf {V}} \!=\! \int _{\Sigma } X_{\mu _1}^{\mu _2} (\alpha ^{\mu _1}_{N-1 \mu _2} - \alpha ^{\mu _2}_{N-1 \mu _1}){\mathbf {V}} \!=\!0. \end{aligned}$$

Hence, we deduce that $\alpha ^{\mu _1}_{N-1 \mu _2} - \alpha ^{\mu _2}_{N-1 \mu _1}=0$. Furthermore, the components of $p_{{\mathcal {T}}}(\alpha )$ satisfy $p_{{\mathcal {T}}}\alpha ^{\mu _1}_{N-1 \mu _2} + p_{{\mathcal {T}}}\alpha ^{\mu _2}_{N-1 \mu _1}=0$. Hence, we conclude $p_{{\mathcal {T}}}\alpha ^{\mu _1}_{N-1 \mu _2}=0$ for all $\mu _1$ and $\mu _2$. Now, consider $\tau$ such that the only nonzero components are $Y_\mu$. Hence, now

$$\begin{aligned} \int _{\Sigma } \tau \alpha&= \int _{\Sigma } \sum _{\mu =1}^{N-2} Y_{\mu } \alpha ^{\mu }_{N-1 \mu }{\mathbf {V}} = \int _{\Sigma } \sum _{\mu =1}^{N-3} Y_{\mu } (\alpha ^{\mu }_{N-1 \mu } - \alpha ^{N-2}_{N-1 N-2}){\mathbf {V}}=0. \end{aligned}$$

By the arbitrariness of $\tau$, we deduce that $\alpha ^{\mu }_{N-1 \mu } - \alpha ^{N-2}_{N-1 N-2}=0$ for each $\mu =1,\dots N-3$. Furthermore, the components of $p_{{\mathcal {T}}}(\alpha )$ satisfy $\sum _{\mu =1}^{N-2} p_{{\mathcal {T}}}(\alpha )^{\mu }_{N-1 \mu }=0$. Hence, we deduce that $p_{{\mathcal {T}}}(\alpha )^{\mu }_{N-1 \mu }=0$ for all $\mu$. This proves the claim. $\square$

### Proof of Lemma 11

Let $\tau \!\in \! {\mathcal {S}}$. Then, we want to prove that $[\tau , e] \!\in \! {{\,\mathrm{Im}\,}}W_{N-3}^{\partial , (1,1)}$. Using the results of Lemma 4, we know that the free components of ${{\,\mathrm{Im}\,}}W_{N-3}^{\partial , (1,1)}$ are

$$\begin{aligned} X_{\mu _1 \dots \mu _{N-2}}^{\mu _1 \dots \mu _{N-2}}, \; X_{\mu _1 \dots \mu _{N-3}\mu _{N-2}}^{\mu _1 \dots \mu _{N-3}\mu _{N-1}} \; \text {and} \; X_{\mu _1 \dots \mu _{N-3}N}^{\mu _1 \dots \mu _{N-3}\mu _{N-2}} \end{aligned}$$

such that $$X_{\mu _1 \dots \mu _{N-3}N}^{\mu _1 \dots \mu _{N-3}\mu _{N-2}}=X_{\mu '_1 \dots \mu '_{N-3}N}^{\mu '_1 \dots \mu '_{N-3}\mu _{N-2}}$$.

From Proposition 8, we deduce that the free components of $\tau \in {\mathcal {S}}$ are

$$\begin{aligned} \tau ^{NN-1 \mu _1 \dots \mu _{N-3}}_{\mu _1 \dots \mu _{N-3}} \; \text {and} \; \tau ^{N N-1 \mu _1 \dots \mu _{N-4} \mu _{N-2}}_{\mu _1 \dots \mu _{N-4}\mu _{N-3}} \end{aligned}$$

such that

$$\begin{aligned} \sum _{\mu _i=1}^{N-2}\tau ^{NN-1 \mu _1 \dots \mu _{N-3}}_{\mu _1 \dots \mu _{N-3}}=0, \\ \tau ^{N N-1 \mu _1 \dots \mu _{N-4} \mu _{N-2}}_{\mu _1 \dots \mu _{N-4}\mu _{N-3}}+ \tau ^{N N-1 \mu _1 \dots \mu _{N-4} \mu _{N-3}}_{\mu _1 \dots \mu _{N-4}\mu _{N-2}}=0. \end{aligned}$$

Recalling that $[\tau , {\tilde{e}}]=0$, we deduce that $[\tau , e]$ has components^26^

$$\begin{aligned}{}[\tau , e]_{\mu _1 \dots \mu _{N-3} N-1}^{\nu _1 \dots \nu _{N-2}}= \tau _{\mu _1 \dots \mu _{N-3}}^{\nu _1 \dots \nu _{N-2} N}. \end{aligned}$$

Plugging into this expression the free components of $\tau$, we get the free components of $[\tau , e]$:

$$\begin{aligned}{}[\tau , e]^{N-1 \mu _1 \dots \mu _{N-3}}_{N-1 \mu _1 \dots \mu _{N-3}} \; \text {and} \; [\tau , e]^{N-1 \mu _1 \dots \mu _{N-4} \mu _{N-2}}_{N-1 \mu _1 \dots \mu _{N-4}\mu _{N-3}} \end{aligned}$$

such that

$$\begin{aligned} \sum _{\mu _i=1}^{N-2}[\tau , e]^{N-1 \mu _1 \dots \mu _{N-3}}_{N-1 \mu _1 \dots \mu _{N-3}}=0, \\ [\tau , e]^{N-1 \mu _1 \dots \mu _{N-4} \mu _{N-2}}_{N-1 \mu _1 \dots \mu _{N-4}\mu _{N-3}}+ [\tau , e]^{N-1 \mu _1 \dots \mu _{N-4} \mu _{N-3}}_{N-1 \mu _1 \dots \mu _{N-4}\mu _{N-2}}=0. \end{aligned}$$

It is straightforward to check that these components are in the image of $W_{N-3}^{\partial , (1,1)}$.

$$\square $$

### Proof of Corollary 12

Using the standard basis, we have that

$$\begin{aligned} (X \wedge e^{N-3})_{i \mu _1 \dots \mu _{N-3}}^{j \mu _1 \dots \mu _{N-3}}&= X_{i}^{j} \text { for } i \ne j \\ (X \wedge e^{N-3})_{ \mu _1 \dots \mu _{N-2}}^{\mu _1 \dots \mu _{N-2}}&= \sum _{\mu } X_{\mu }^{\mu } \text { with } \mu \in \{\mu _1 \dots \mu _{N-2}\}. \end{aligned}$$

Comparing these expressions with the ones in the proof of Lemma 11, we deduce that $[W_{N-3}^{-1}([\tau ,e])]_{\mu _1}^{\mu _2} \propto X_{\mu _1}^{\mu _2}$ and that

$$\begin{aligned} \sum _{\mu =1, \mu \ne \nu }^{N-1} [W_{N-3}^{-1}([\tau ,e])]_{\mu }^{\mu } = Y_{\nu }. \end{aligned}$$

Summing for $\nu =1 \dots N-1$ and remembering that $Y_{N-1}=0$ and that $\sum _\nu Y_\nu =0$, we deduce the claim. $\square$

### Lemma 50

Let *D* be an invertible matrix such that the inverse does not contain derivatives and let *B* some matrix proportional to an odd parameter $\lambda$ and not containing derivatives. Then, $BD^{-1}B^T=0$.

### Proof

The key point of the proof is that every term containing $\lambda ^2$ vanishes since $\lambda$ is an odd quantity. Now, by hypothesis, every term in $B D^{-1} B^T$ does not contain derivatives and is quadratic in $\lambda$, hence it vanishes. $\square$

## Footnotes

1 By number of local degrees of freedom, we mean the rank of the phase space as a $C^\infty$-module (ignoring global degrees). In the spacelike or timelike cases, one also usually speaks of the number of local physical degrees of freedom meaning by this half the rank of the reduced phase space (i.e. the rank of the configuration space).

2 In a language more common in the physics literature, using index notation, we can say that we can equip an element in $\Omega ^{i,j}$ with *i* contravariant indices (antisymmetrized in the cotangent space of *M* and *j* antisymmetrized indices in ${\mathcal {V}}$.

3 Using index notation this map corresponds to taking antisymmetrization in both set of indices. Note also that the combinatorial factor arising in such operation is absorbed in the definition of wedge product and will not appear in formulas without indices.

4 Note that the quantities appearing in this integral are elements of $\Omega ^{N,N}$ which can be canonically identified with the space of densities on *M*, hence this integral is well defined. This same observation holds for every integral appearing in the paper. See [5] for a detailed explanation.

5 For differential forms, we might as well speak of pullback with respect to the inclusion of the boundary in the bulk.

6 i.e. a closed, non-degenerate two-form.

7 We will use the same symbols for the fields on the bulk and the corresponding pullbacks (or restrictions) to the boundary.

8 More precisely, note that, by the non-degeneracy condition on *e*, at each $u\in \Sigma$ we have that $e(T_u\Sigma )$ is an oriented, three-dimensional subspace of ${\mathcal {V}}_u$. The field $e_n$ is chosen so that $e_n(u)$ is transversal to $e(T_u\Sigma )$ and compatible with the orientation. Equivalently, if we pick local coordinates $(x^1,x^2,x^3)$ around *u* and expand $e=e_1 dx^1+e_2 dx^2+e_3 dx^3$, then we require that $(e_1(u),e_2(u),e_3(u),e_n(u))$ be a frame for ${\mathcal {V}}_u$ at each $u\in \Sigma$.

9 Note that this additional condition on the boundary fields is not required for the description of the boundary structure. However, it is useful for fixing a representative of the equivalence class $[\omega ]$.

10 We thank M. Schiavina for the helpful discussion about the form of this constraint (and its name).

11 Other sign conventions are possible, for example, the one with separate degrees. Different conventions lead to isomorphic vector spaces but not isomorphic algebras.

12 Later we will also consider elements with *ghost number*. This means that we consider an additional ${\mathbb {Z}}$-grading and the total degree will be the sum of all the degrees.

13 The fact that the required condition is sufficient and well defined will be analysed later in Lemma 48.

14 For example, it is possible to obtain an explicit expression for the complement in the following way. Choose an arbitrary Riemannian metric on $\Sigma$ and extend it to $\Omega ^{2,1}$. Then, it is possible to view *J* as the orthogonal complement of ${{\,\mathrm{Im}\,}}\varrho ^{(1,2)} |_{\mathrm {Ker} W_{N-3}^{\partial , (1,2)}}$ in $\Omega _{\partial }^{2,1}$ with respect to this Riemannian metric. This approach will be used in “Appendix C” to prove the lemmas and proposition below with the diagonal Riemannian metric.

15 In order to define these projections, we may proceed as in footnote 14 and define an orthogonal complement of these spaces and subsequently use the corresponding orthogonal projections.

16 Such quantities are also sometimes referred to as Grassmann variables.

17 The critical locus of these constraints does not depend on $\omega _0$, since it appears in (17b17c) in combination with an expression already present in (17a).

18 It is useful to stress here the differences in the notation between the first constraint and the Lie derivative. The first is denoted with an italic *L*, while the second with a calligraphic ${\mathcal {L}}$.

19 Starting from the definition of ${\mathcal {K}}$ in (9), it is a straightforward check that this last equation fixes the components of $\omega \in \mathrm {Ker}{( W_{N-3}^{\partial ,(1,2)})}$ not included in (20b). Indeed, the elements of $\omega \in \mathrm {Ker}{(\rho )}$ are the ones that no longer appear in the structural constraints in the degenerate case opposed to the non-degenerate one.

20 As before the notation [1] denotes that $\tau$ is an odd quantity.

21 Note that, in order to avoid cumbersome notation, we will from now on avoid to write all the indices of the inverse functions of $W_{\bullet }^{\partial ,(\bullet ,\bullet )}$ and of ${\widetilde{\rho }}^{(\bullet ,\bullet )}$.

22 Using the lemmas in Sect. 2, it is possible to prove that all the nonzero components of $\sigma$ are in the direction of $e_n$.
